# Accuracy of CSF tap test and lumbar infusion test in predicting shunt response in idiopathic normal pressure hydrocephalus: a systematic review and meta-analysis

**DOI:** 10.3389/fneur.2026.1908328

**Published:** 2026-10-12

**Authors:** Aminu Aliyar, Deepa Dash, Alfonso Fasano, Manya Prasad, Vikas Dhikav, Aparna Wagle Shukla, Ashish Datt Upadhyay, Soaham Desai, Sagar Poudel, Pramod Kumar Pal, Arunmozhimaran Elavarasi

**Affiliations:** 1 Department of Neurology, All India Institute of Medical Sciences, New Delhi, India; 2 Department of Clinical Neurologic Sciences, University of Western Ontario, London, ON, Canada; 3 Department of Biomedical Sciences, Humanitas University, Pieve Emanuele, Milan, Italy; 4 IRCCS Humanitas Research Hospital, Rozzano, Milan, Italy; 5 Center for Community Medicine, All India Institute of Medical Sciences, New Delhi, India; 6 Center for Evidence for Guidelines, Department of Health Research, MoHFW, Govt of India, New Delhi, India; 7 Department of Neurology, Fixel Institute for Neurological Diseases, University of Florida, Gainesville, FL, United States; 8 Clinical Research Unit, All India Institute of Medical Sciences, New Delhi, India; 9 Department of Neurology, Pramukhswami Medical College, Karamsad, Anand, India; 10 Department of Medicine, All India Institute of Medical Sciences, New Delhi, India; 11 Department of Neurology, National Institute of Mental Health and Neurosciences, Bengaluru, India

**Keywords:** CSF tap test, lumbar infusion test, normal pressure hydrocephalus, NPH, diagnostic accuracy, sensitivity, specificity

## Abstract

**Background:**

Idiopathic normal pressure hydrocephalus (iNPH) presents with gait disturbance, cognitive impairment, and urinary incontinence. The cerebrospinal fluid tap test (CSF-TT) and lumbar infusion test (LIT) are commonly used to predict postoperative improvement after shunt surgery; however, their validity remains debated.

**Methods:**

We performed a systematic review and meta-analysis to assess the sensitivity and specificity of CSF-TT and LIT. The protocol was registered in PROSPERO (CRD42023454502). Reporting followed the PRISMA-DTA guidelines, and the certainty of evidence was assessed using the GRADE approach.

**Results:**

Of 1,955 studies, 15 were included, reporting on 721 CSF-TT and 393 LIT patients, with shunt surgery as the reference standard. Nine CSF-TT studies (*n* = 700) and five LIT studies (*n* = 368) contributed to the primary meta-analysis. Considerable heterogeneity existed in test protocols, timing of assessment, and outcome measures. Several studies were retrospective with a high risk of bias. The pooled estimates were derived from a limited number of heterogeneous studies. Using a bivariate random-effects model, pooled sensitivity and specificity of CSF-TT were 64.1% (95% *CI* 49.2–76.6%, *I*^2^ 81.3%) and 54.8% (95% *CI* 41.8–67.2%, *I*^2^ 59.7%), respectively. The certainty of evidence was very low for sensitivity due to bias, inconsistency, and imprecision, and low for poor specificity due to bias and inconsistency. LIT showed a pooled sensitivity of 77.5% (95% *CI* 70.2–83.4%, *I*^2^ 14.7%) and a specificity of 28.3% (95% *CI* 19.0–39.9%, *I*^2^ 4.6%), with moderate certainty for sensitivity and moderate certainty for poor specificity.

**Conclusion:**

Both CSF-TT and LIT show poor specificity for predicting shunt responsiveness in iNPH, with low-certainty evidence for CSF-TT and moderate-certainty evidence for LIT. A positive result from either test should not be used in isolation to guide surgical decisions. Standardized prospective studies with clearly defined patient selection criteria and outcome measures are needed to establish the true diagnostic value of these tests.

**Systematic review registration:**

https://www.crd.york.ac.uk/PROSPERO/view/CRD42023454502.

## Background

Normal pressure hydrocephalus (NPH) was first described by Hakim and Adams in 1965. It is characterized by the triad of gait disorder, cognitive impairment, and urinary incontinence. It is a potentially treatable disorder, with randomized controlled trials to support the efficacy of surgery (1, 2). The prevalence of possible idiopathic NPH (iNPH) ranges from 10/100,000 to 29/100,000, with age-specific rates varying from 3.3/100,000 in individuals aged 50–59 to 5.9% in those aged 80 years or older (3). Several imaging features, such as the callosal angle, Evan's index, and disproportionately enlarged subarachnoid spaces (DESH), have been described that help identify patients for ancillary testing. Patients with suspected iNPH undergo a CSF tap test (CSF-TT) and/or lumbar infusion test (LIT), with serial monitoring of gait scores and cognitive function. Improvement in symptoms is considered a harbinger of successful symptom control following CSF shunting, such as ventriculoperitoneal (VP) or lumbo-peritoneal shunting. External lumbar drainage (ELD) is another minimally invasive method for CSF diversion; however, since it requires hospital admission and carries some risks (e.g., infection), it is not commonly performed in the preoperative phase.

While some guidelines acknowledge that the CSF-TT has poor sensitivity (4, 5), they claim its specificity is quite high (5). There are conflicting opinions regarding their predictive power and validity (6, 7). The Japanese guidelines advise clinicians that even if ancillary tests are negative, patients may still be referred for shunt surgery if other criteria, such as clinical and radiological features, are consistent with the diagnosis. Despite these recommendations, there is wide variation in clinical practice, and several institutions, such as the UK-NHS (8) and the Canadian Health System (9), use CSF-TT results to decide who undergoes a definitive surgical procedure. The optimal cut-offs and timing of assessment following CSF-TT to predict improvement after shunting are unknown (10, 11). Despite variable endorsement in international guidelines, evidence supporting standardized protocols for these tests remains limited. Since there is clinical equipoise regarding the diagnostic accuracy of CSF-TT and LIT for predicting clinical shunt response in patients diagnosed with iNPH, this systematic review aims to synthesize the diagnostic accuracy of these tests, with a focus on methodological heterogeneity and risk of bias across studies. In NPH, a “definite” diagnosis is established only when the patient improves following shunt surgery. Consequently, the patient's postoperative outcome serves as the reference standard, meaning that prognosis directly determines the diagnostic accuracy of tests performed before shunting.

## Methods

We conducted a systematic review and meta-analysis to assess the sensitivity and specificity of CSF-TT and LIT for predicting outcomes after shunt surgery in patients with iNPH. The protocol for this review was registered with PROSPERO under the registration number CRD42023454502. We adhered to the Preferred Reporting Items for Systematic Reviews and Meta-Analyses of Diagnostic Test Accuracy Studies (PRISMA-DTA) guidelines for reporting this systematic review (Supplementary file 1).

We included studies of patients with idiopathic NPH, defined according to clearly described clinico-radiologic criteria in the individual studies, who underwent diagnostic testing with a large-volume CSF-TT or LIT, followed by VP or lumboperitoneal shunt surgery, regardless of the results of these preoperative ancillary tests. These studies reported clinico-radiological details and/or functional outcomes of patients after surgery and were published in English. To avoid circular reasoning, we excluded studies that reported results of surgery in patients who were operated on based on the results of predictive tests (i.e., CSF-TT or LIT) and those with incomplete clinico-radiological details or lacking objectively quantified outcomes following surgery. We developed a search strategy that included keywords and other terms to address the diagnostic aspects of iNPH. The search strategy was approved by all authors, following several iterations to finalize the approach (Supplementary file 2). We searched MEDLINE (Ovid), Embase, Cochrane CENTRAL, and conducted a PubMed search for studies not yet indexed in MEDLINE on July 10, 2026. We also searched the reference lists of all included studies and relevant systematic reviews for additional references, and the Scopus database for studies that met the inclusion criteria listed above. All studies obtained through electronic search were stored in Zotero reference management software (version 9.0.6, Corporation for Digital Scholarship, Virginia, USA) and then imported into Covidence systematic review software (Veritas Health Innovation, Melbourne, Australia), where the references were de-duplicated, screened, and full-text review, data extraction, and quality assessment were done. A pair of reviewers (AA and AE) independently screened titles and abstracts, reviewed the full texts of potentially eligible studies to determine the final eligible studies, and abstracted data. We abstracted the last name of the first author, year of publication, country, and hospital, as well as population, interventions, and outcomes, into prespecified abstraction forms. Disagreements between the reviewers were resolved by discussion or by a third reviewer (SD).

### Thresholds for index test positivity and definitions for the reference standard

The thresholds and definitions used in the individual studies were used to analyze diagnostic accuracy. Where a study reported multiple thresholds, domains, or assessment time points, we applied a consistent selection hierarchy: the composite outcome score (combining gait, urinary, and cognitive domains) was used as the primary outcome where available; where no composite score was reported, the gait-based outcome (e.g., the TUG test) was used as a fallback, since gait was the most consistently reported domain across studies; and where additional gait-related parameters were reported alongside the standard TUG test, the standard TUG test was retained to preserve comparability across studies. In LIT, we designated 12 mmHg/ml/min as the index test Rout threshold for the primary analysis, as it is the most widely accepted threshold. Where a study reported a related but non-identical cut-off, we pooled them together pragmatically. Studies using a substantially different cut-off were excluded from the primary analysis. This selection hierarchy was applied *post hoc* during data extraction and was not prespecified in the PROSPERO protocol.

### Risk of bias assessment

Quality Assessment of Diagnostic Accuracy Studies 2 (QUADAS-2) framework (12) was employed to assess the risk of bias explored across four domains: patient selection (3 questions), index test (2 questions), reference standard (2 questions), and flow and timing (4 questions), which were answered as “yes,” “no,” or “unclear” based on the data reported in the individual studies. The pair of reviewers (AA and AE) assessed the risk of bias based on the answers to the questions as follows: low risk if all answers were “yes”; unclear risk of bias if at least one question was answered as “unclear”; and high risk of bias if at least one question was answered as “no.” For the first three domains, the applicability concerns were assessed as low, unknown, or high risk if the population, index test, or reference standard did not align with the review question. Disagreements between the reviewers were resolved through discussion and, in some cases, by the third reviewer (SD).

### Statistical analysis

Sensitivity and specificity analyses were performed by extracting the numbers of patients with and without a response to CSF-TT or LIT, as well as the numbers who improved and did not improve following surgery. We conducted a meta-analysis of diagnostic test accuracy using the metadta/metandi command in Stata version 17.0 (StataCorp LLC, College Station, TX, USA). These commands implement a bivariate random-effects model that jointly analyzes sensitivity and specificity, accounting for their correlation and between-study variability (13–15). True positives, false positives, false negatives, and true negatives were extracted from each study to estimate pooled sensitivity, specificity, diagnostic odds ratio, and to construct the hierarchical summary receiver operating characteristic (hSROC) curve. The τ^2^ statistic and *I*^2^ were used to assess heterogeneity. The positive (LR+) and negative (LR–) likelihood ratios were calculated from the point estimates of sensitivity and specificity, and the confidence intervals were computed assuming asymptotic normality using a log transformation, incorporating the correlation between sensitivity and specificity estimated by the bivariate model. Post-test probabilities for positive and negative index test results were calculated by applying pooled sensitivity and specificity to pre-test probabilities of 50%, 60%, and 80%, with confidence intervals derived by substituting the corresponding sensitivity and specificity confidence limits.

No prespecified sensitivity analysis was planned for risk-of-bias assessment. Studies enrolling patients with confirmed non-iNPH populations, such as secondary NPH or atypical NPH with brain atrophy, or those using different cut-offs were excluded from the primary analysis, consistent with the prespecified inclusion criteria. A *post hoc* sensitivity analysis including these studies was performed to assess their influence on the pooled estimates. Another sensitivity analysis included studies that reported the TUG test as one of the index tests. We also performed a leave-one-out sensitivity analysis iteratively refitting the bivariate model while excluding one study at a time and comparing the resulting pooled sensitivity, specificity, and correlation estimates across iterations. Univariate random-effects meta-analyses of sensitivity and specificity were also performed separately using the metaprop command, which fits a generalized linear mixed binomial-normal model to each proportion independently, to assess the consistency of the pooled estimates when the correlation between sensitivity and specificity is not jointly modeled. To assess the sensitivity of the LR+ and LR– confidence intervals to the boundary correlation estimate in the primary bivariate model, we performed two complementary analyses. First, we recalculated LR+ and LR– using the univariate sensitivity and specificity estimates obtained separately, treating them as independent and propagating uncertainty via the delta method on the logit scale. Second, as a confirmatory analysis, we refitted the bivariate model directly on the study-level data with the between-study correlation constrained to zero (an independent covariance structure) rather than estimated freely (xtmelogit, Stata version 17). In both these analyses, confidence intervals for LR+ and LR– were derived using the delta method applied to the relevant logit-scale coefficients and their covariance matrix.

### Certainty of evidence

We used the Grading of Recommendations Assessment, Development and Evaluation (GRADE) approach to assess the certainty of evidence for each primary outcome, considering factors such as risk of bias, inconsistency, indirectness, imprecision, and publication bias. To address grading inconsistency and imprecision, we set an exploratory, *post hoc* decision threshold of 70% for both sensitivity and specificity as a pragmatic benchmark for clinical usefulness. Since shunt surgery carries a relatively low frequency of complications, the harm of offering surgery to a non-responder may be comparatively limited, whereas missing a true responder could mean forgoing a potentially beneficial intervention. However, the relative consequences of false-positive and false-negative results depend on factors such as surgical risk, comorbidities, expected benefit, and patient preference. High sensitivity is necessary to avoid missing true responders; however, imaging biomarkers are highly sensitive (16), and current guidelines do not recommend withholding surgery based solely on negative ancillary test results. The more clinically relevant question is therefore whether the pooled specificity and its confidence intervals are sufficient to support ruling in shunt responsiveness, which is why we applied a 70% threshold to evaluate this. As a *post hoc* check, we also examined whether adopting a more permissive sensitivity threshold of 50% would alter our certainty ratings.

## Results

The electronic search identified 1,955 studies, and 486 duplicates were removed. A total of 1,469 articles were screened for eligibility as per our inclusion and exclusion criteria, and 92 studies were selected for full-text review. We extracted data from 15 studies that reported the results of the index tests and reference standards as per the review question (Figure 1). Ten studies reported the diagnostic accuracy of the CSF-TT, and six studies reported the diagnostic accuracy of LITs in NPH. The multicentric cohort study by Wikkelsö et al. (17) contributed to both the CSF-TT and LIT pooled estimates.

**Figure 1:**
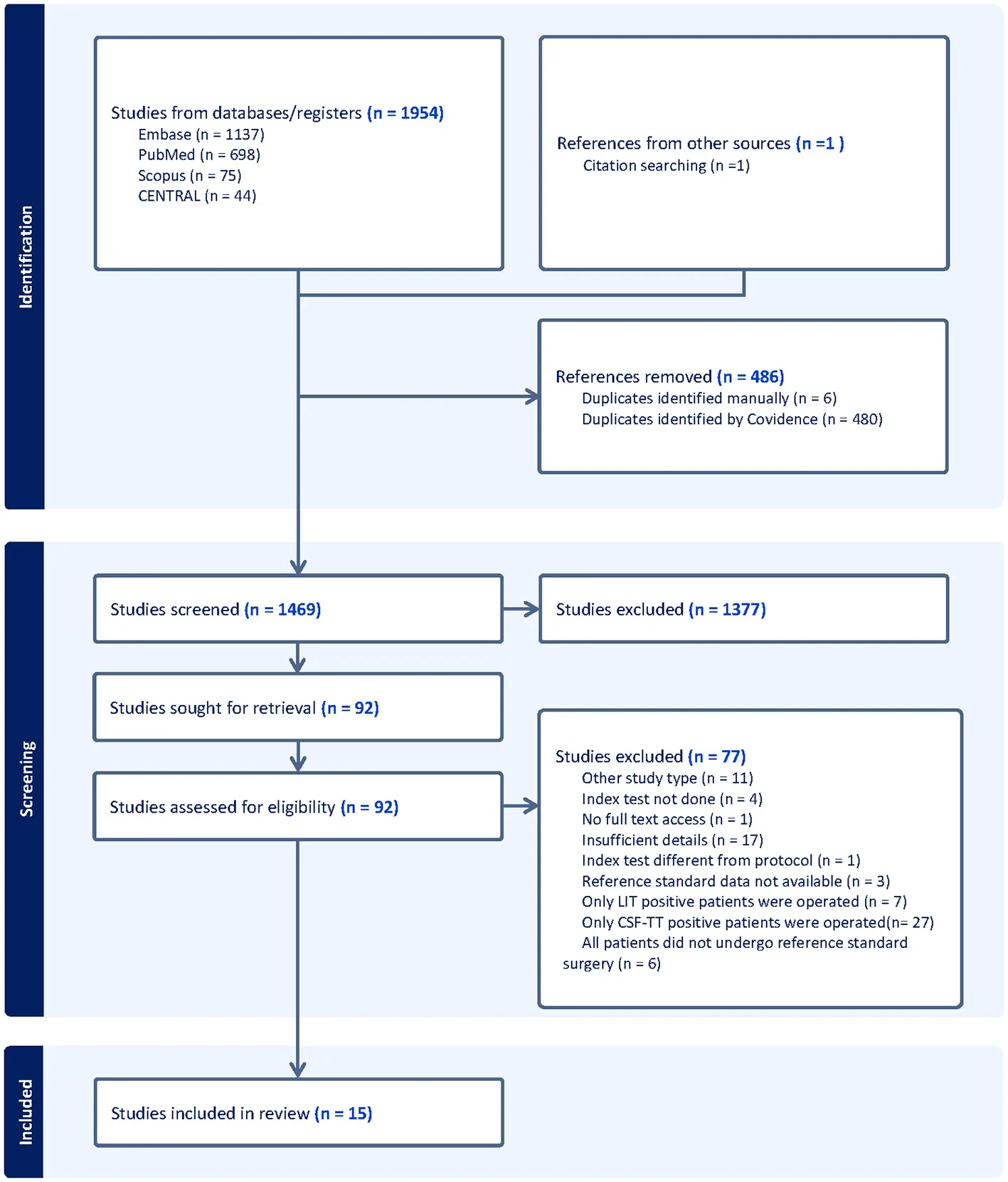
PRISMA flow diagram of the study selection process.

### CSF tap test and post-shunt outcomes

We identified ten cohort studies (17–26), comprising three prospective, four retrospective, one ambispective, and two with an unknown direction of data collection (*n* = 721). The characteristics of these studies have been described in detail in Supplementary Table 1. The mean ages of the patients ranged from 63 to 78.8 years. Gait dysfunction varied from 84% to 100%, urinary dysfunction between 60 and 100%, and cognitive dysfunction between 72 and 100%. The duration of symptoms ranged from 1 month to 11 years across six studies; however, in four studies, it was not reported. The radiologic findings, selection criteria for shunting, and the time from evaluation to the therapeutic procedure varied significantly across studies, as shown in Table 1.

**Table 1:** CSF tap test: technique, assessment, tap responsiveness, shunt responsiveness, and diagnostic accuracy.

Study ID; country	CSF tap test procedure and criteria for positivity (index test protocol)	Reference standard (VP shunt responsiveness): timing of surgery, outcome measures, and criteria for shunt responsiveness	Diagnostic accuracy measures
1. Wikkelsö et al. (18); Sweden	CSF tap test procedure: lumbar puncture of 50 cc CSF Tests: psychometric testing, number of steps to walk 18 m Assessment: before the tap test and 2 h after the tap test Criteria for a positive tap test: The assessment was conducted 2 h after the tap test. Identical forms 25 %; Bingley's memory test 25%; reaction time test 5%; and walking test 5%	Timing of shunt surgery after CSF tap test: NR Postoperative assessment was performed 3–6 months after shunt placement. Criteria for shunt response: NR	Walk test: sensitivity: 89; specificity: 100 Memory test: sensitivity:77; specificity: 75
2. Ishikawa et al. (19); Japan	CSF tap test procedure: a lumbar tap with the removal of 30 ml of CSF Tests: iNPH grading scale; the mini-mental state Examination (MMSE), and the 3-m timed “up and go” test (TUG). Assessment: Change in gait 1–2 days after tap, cognition, and urination at 1 week after tap Criteria for a positive tap test: an improvement of one point or more on the iNPH grading scale (in each domain and its total), a more than 10% improvement in time on the TUG, or a more than 3-point improvement in the MMSE; Improvement in any of the total scores of iNPHGS, TUG, or MMSE was defined as positive, with an additional variable of tap-any	Timing of shunt surgery after CSF tap test: within 2 months Assessment: at 12 months Shunt responder: improvement of one point or more on the modified Rankin scale	Gait scale: sensitivity: 51.3 Specificity: 80; TUG sensitivity: 34.2; specificity: 73.6; urinary scale sensitivity: 37.5; specificity: 85 MMSE Sensitivity: 63.8; specificity: 30 Total gait scale sensitivity: 71.3; specificity: 65 Tap any: sensitivity 92.5; specificity 20
3. Wikkelsö et al. (17)	CSF tap test procedure: a CSF TT of 50 ml was performed at approximately 09:00 h Tests: gait—number of steps and the time needed to walk 10 m at free speed Psychometric performance using the Grooved Pegboard (Lafayette Instrument Co.), which measures manual dexterity as the time to fit 25 pegs into holes with randomly positioned slots, and the Stroop test Assessment: 3 h after drainage of cerebrospinal fluid Criteria for a positive tap test: response to the CSF TT was expressed as the mean percentage change across all motor and psychometric tests compared with results obtained 24 h previously (the day before the combined CSF dynamic test). A mean increase of 5% was considered significant	Timing of shunt surgery after CSF tap test: NR Assessment: at 12 months postoperatively Shunt responder: Improvement in the iNPH Scale was defined as an increase of 5 points or more, and in the mRS as a decrease of 1 point.	Improvement in motor and psychometric scores (>5%) Sensitivity: 52% Specificity: 59%
4. Yamada et al. (20); Japan	CSF tap test procedure: 30 ml of CSF via a lumbar puncture Tests: iNPH grading scale, timed 3-m up-and-go test (TUG), and 3-m reciprocating walking test (RWT) Assessment: gait was evaluated within 24 h, and cognitive impairment within 7 days Criteria for positive tap test: clinical improvement in gait was assessed by ≥1 point improvement of the gait domain of the iNPHGS, and combined gait evaluation was defined as ≥1 point improvement of the gait domain of the iNPHGS combined with ≥ 10% shortening of the time on the TUG or 3-m RWT Clinical improvement of cognitive impairment was assessed as ≥1-point improvement in the cognitive domain of the iNPHGS. A combined cognitive evaluation was defined as a ≥ 1-point improvement in the cognitive domain of the iNPHGS, combined with a ≥ 3-point improvement on the MMSE or a ≥ 2-point improvement on the FAB In addition, a ≥1-point improvement in the modified Rankin Scale and the total iNPHGS was used to assess general improvement	Timing of shunt surgery after CSF tap test: within 3 months Assessment: 12 months Criteria for shunt responsiveness: improvement of the outcome variables at 12 months after the surgery	iNPHGS sensitivity: 61.3% 19/31; Specificity: 64.7% 33/51 mRS Sensitivity 27.5 Specificity 93.3
5. Liu et al. (21); China	CSF tap test procedure: 30 ml of CSF was removed via lumbar tap Assessment: before and 8, 24, 72 h after LP Test: (1) walking tests:10-m walking time, 10-m walking steps, and 5-m timed up and go test (TUG) time For the TUG, the amount of time required for a patient to rise from an armchair, walk 5 m, turn, walk back to the chair, and sit down. Again, at maximum speed was recorded. For 10Ti and 10St tests, the patient was also asked to walk at maximum speed. Each assessment was performed twice, and the average time and steps were calculated Cognitive assessment: the brief executive battery included the symbol-digit modalities test, trail making test A, and STROOP-C. The composite *z*-scores of the brief executive function battery were calculated by averaging the *z*-scores of the three tests according to the Chinese norm Criteria for positive tap test: CSF TT responders: (1) the time or number of steps in the walking tests were reduced by 10% at a minimum of one time point after the CSF TT; or (2) the executive function composite *z*-score was improved by more than 20% (any time after the tap test, 8 h, 24 h, and 72 h)	The shunt was done within 1 month of TT Assessment: 34 ± 26 months Criteria for shunt responsiveness: improvement reported by the patient or caregiver, accompanied by at least a one-point increase or more in the Japanese iNPHGS	24 h testing: TUG sensitivity 35.7%; specificity 66.7% 10 m time test sensitivity 37.5%, specificity 85.7% Composite *Z*-score: sensitivity 72.7%, specificity 80% Cumulative TUG sensitivity 57%, specificity 43%, composite *Z*-score sensitivity 82%, specificity 60%
6. Rydja et al. (22); Sweden	CSF tap test procedure: a neurologist performed the LP with the patient in a lateral position. A spinal fluid manometer was used to measure lumbar cerebrospinal fluid (CSF) pressure for 30 s before CSF removal Assessment: approximately 3 h after the LP Tests: gait and balance domains in the iNPH scale Criteria for positive tap test: outcomes assessed 3 h after the tap test. CSF TT responders with a positive outcome (≥5 points) in the gait domain on the Hellström iNPH scale	Surgery was done 126 (102–159) days after baseline assessment Assessment: 3 months following surgery Criteria for shunt responsiveness: A limit of ≥5 points for the total Hellström iNPH scale is proposed as an improvement after surgery	Gait domain iNPH scale as the outcome in TT and total iNPH as postoperative outcome—sensitivity: 68.1%; specificity: 52% Gait domain as an outcome in the CSF TT and the same outcome postoperatively: sensitivity 72%, specificity 52%
7. Kameda et al. (23); Japan	CSF tap test procedure: 30 ml of CSF was tapped Assessment: If the tap test was performed as an inpatient investigation, the TUG assessment was mainly performed 1 or 3 days after the tap test. If the tap test was performed as an outpatient investigation, the TUG was assessed immediately after the tap test and at the next outpatient visit, up to 2 weeks later. If TUG was evaluated multiple times after the tap test, the best one was used Tests: the functional gait assessment (FGA) is a 10-item evaluation of gait. Following the tap test, patients were asked to indicate whether they perceived a change in their gait using a Global Rating of Change (GRC) scale Criteria for a positive tap test to calculate diagnostic accuracy measures: FGA improvement of 4 or more, GRC improvement of 2 or more, TUG improvement of 10% or more	The timing of the LP shunt after the tap test is not mentioned Criteria for shunt responsiveness: Improvement in the Japanese iNPH score, cut-offs, and the timing of post-surgery assessment are not specified	TUG: sensitivity 23%, specificity 71% FGA: sensitivity 83%, specificity 17% GRC: sensitivity 70%; Specificity: 25% Missing outcomes in FGA and GRC
8. van Bilsen et al. (24); Netherlands	CSF tap test procedure: 50 ml tap test Criteria for positive tap test: no cut-offs for tap test responsiveness have been reported	The timing of surgery after the index test was not mentioned. Median follow-up duration was 6 months Improvement after shunting was defined as a minimum one-point improvement in the NPH score Median time to surgery: 1 month in responders (1–2);1.5 months in non-responders (1–3.5)	Sensitivity: 77 Specificity: 25
9. Gao et al. (25); China	CSF tap test procedure: not reported Assessment: gait was assessed using the 10 m walk test; cognition was assessed with the mini-mental state Examination; Urinary incontinence was subjectively assessed by patients on a scale from 0 to 10, indicating the severity from normal to severe. An identical assessment was performed 24–72 h later following the tap test Criteria for positive tap test: At least one of the following: (a) ≥20% improvement in time or steps of the 10 m timed walk test and/or (b) ≥10% improvement in MMSE score and/or (c) an improvement of ≥1 point in Urinary Incontinence score	Timing of surgery after the index test: not reported Median follow-up duration: 6–12 months Criteria for shunt responsiveness: Krauss index at a follow-up of 6–12 months, where responders were defined as those with a Krauss index ≥ 0.5	Sensitivity: 63.5% Specificity:60%
10. Poudel et al. (26); India	CSF tap test: 30–50 ml of CSF drained after baseline assessment with Dutch gait scale, gait, cognitive, and urinary scales using iNPH scale, modified Rankin scale Assessment: all baseline assessments were repeated at 24 h following the CSF tap test Criteria for a positive tap test: reduction of at least one point on the modified Rankin scale at 24 h post CSF tap assessment	Timing of surgery after the index test: Median follow-up duration: 24 ± 2 weeks Criteria for shunt responsiveness: at least one point reduction in the modified Rankin scale at 24-week follow-up	Sensitivity: 53.3% Specificity: 66.7%

The clinical protocols applied for the tap test varied widely, and the clinical tests used differed and are described in detail in Table 1. The volume of CSF tapped during the tap test ranged from 30 ml (19–21, 23) to 50 ml (17, 18, 24). Poudel et al. (26) used a range of 30–50 ml. Improvements were evaluated as early as 2 h after the tap (18) to as late as 1 week for cognitive and urinary symptoms (19, 20). Liu et al. (21) performed serial assessments at 8, 24, and 72 h.

Several different clinical assessment tools: 10-m walking test, Bingley's memory test, reaction time test, MMSE, timed 3-m timed up-and-go test (TUG) and 3-m reciprocating walking test (RWT), iNPH grading system (iNPHGS), Grooved Pegboard (Lafayette Instrument Co, Lafayette, IN), Stroop Test, Symbol-Digit Modalities test, and Trail Making Test A, were used across various studies. Van Bilsen et al. (24) did not specify the criteria for a positive tap test, though they reported the number of patients with a positive test. The timing of shunt surgery after tap tests was either not mentioned or variable between the included studies. Similar heterogeneities existed in the criteria used to define improvement and in the timing of post-surgical outcome assessment (Table 1). The CSF-TT was not employed in the decision-making process in any of these studies. Wikkelsö et al. (18) included patients with secondary NPH and was therefore excluded from the primary analysis.

Nine studies (17, 19–26), including 700 patients, were included in the bivariate random-effects meta-analysis evaluating the diagnostic accuracy of the CSF-TT for predicting shunt responsiveness. The 2x2 contingency table for each included study is presented in Supplementary Table 2. The pooled sensitivity was 64.1% (95% *CI*, 49.2–76.6%) and the pooled specificity was 54.8% (95% *CI*, 41.8–67.2%; Figure 2), as also depicted by the hierarchical summary receiver operating characteristic curve (hSROC) in the Supplementary Figure 1. Substantial between-study heterogeneity was observed for both sensitivity (τ^2^ = 0.73, *I*^2^ = 81.3%) and specificity (τ^2^ = 0.48, *I*^2^ = 59.7%). The generalized *I*^2^ statistic, reflecting overall heterogeneity across the joint bivariate distribution of sensitivity and specificity, was 0.02 with a tau^2^ of 0.00. The covariance between sensitivity and specificity was −0.57, with a correlation coefficient of −1.00. The bivariate model was fitted using adaptive Gauss–Hermite quadrature with five integration points. During the initial phase refining starting values, transient non-concavity was observed at two of four iterations; this resolved during the subsequent gradient-based optimization phase, which converged smoothly over four iterations to a final log likelihood of −49.15, with no non-concavity at the final solution.

**Figure 2:**
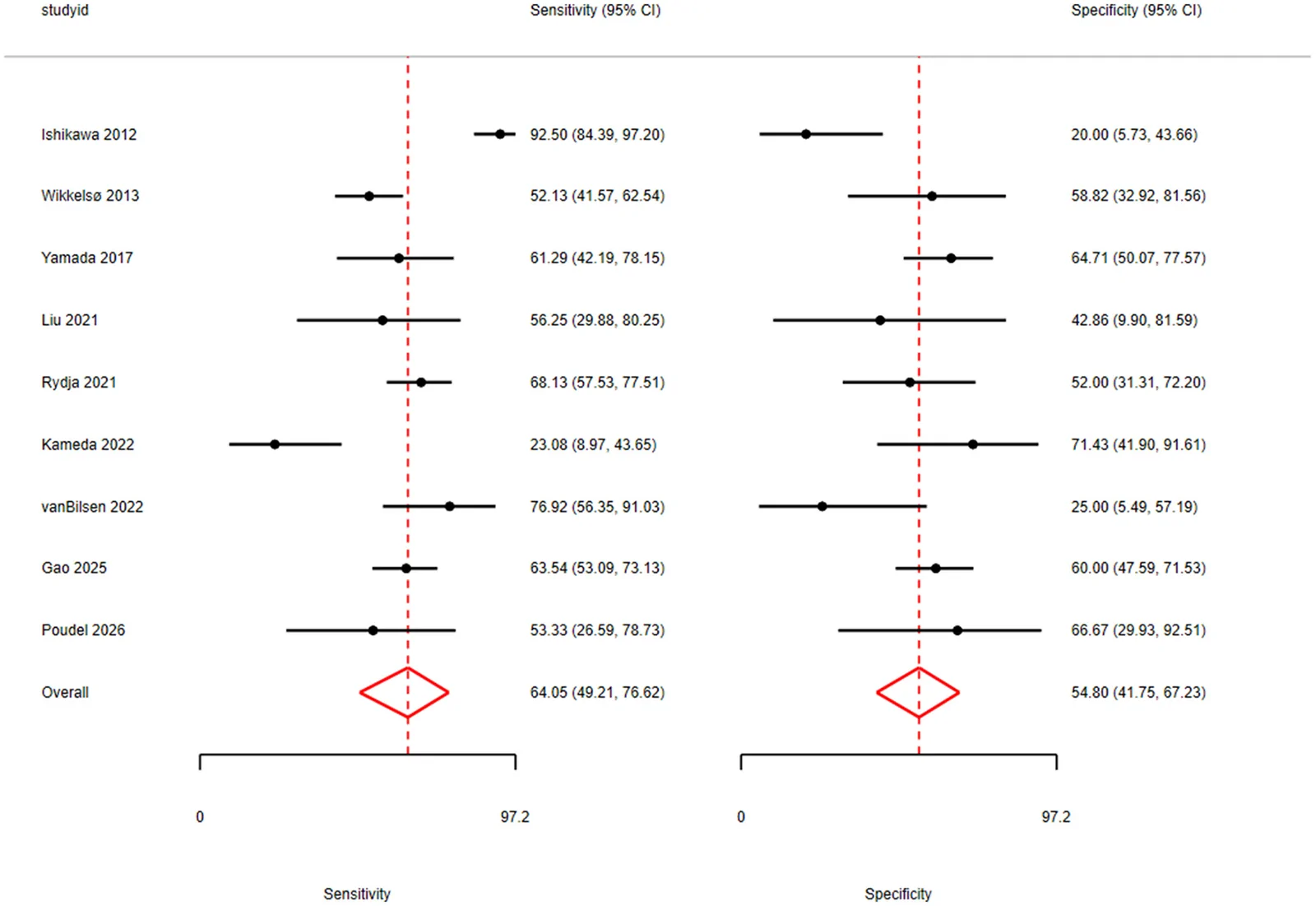
Forest plot showing the pooled sensitivity and specificity of the CSF tap test (CSF-TT).

### Lumbar infusion tests and post-shunt outcomes

Six studies with 393 patients were included (17, 27–31). Three studies were prospective cohort studies, two were retrospective cohort studies, and one had an unknown direction of data collection. The details of these studies are described in Table 2 and Supplementary Table 3. The mean ages ranged from 60.4 to 74 years. Only one study (29) reported clinical symptoms, with gait dysfunction observed in 94% and urinary dysfunction in 88.2% of patients. The remaining studies did not report the proportion of individuals with gait, urinary, or cognitive impairment. As in studies reporting CSF-TT, protocols varied widely.

**Table 2:** Lumbar infusion test: technique, assessment, shunt responsiveness, and diagnostic accuracy.

Study ID; country	Lumbar infusion test: procedure and cut-offs for positivity	VP shunt timing, outcomes assessed, and criteria for shunt responsiveness	Diagnostic accuracy measures
Boon et al. (27); Netherlands	LIT protocol: with the patient placed in the lateral recumbent position, a 19-gauge lumbar needle was connected to a pressure monitor and a fluid-filled infusion system. First, the baseline CSF pressure was measured. Once this pressure remained constant for at least 5 min, saline was infused at a constant rate of 1.4 to 1.6 ml/min until a stable pressure plateau was reached or the pressure exceeded 50 mm Hg. In the latter case, the infusion was repeated at a lower infusion rate. The Rout was calculated as the difference between the plateau and baseline pressures divided by the infusion rate, in mmHg/ml/min Criteria for a positive LIT: multiple Rout values tested for diagnostic accuracy, not prespecified	Timing of shunt surgery: not reported Time to outcome assessment: 1 month following surgery Changes in gait and continence scores were evaluated separately using Boon's NPH scale. Criteria for shunt responsiveness: Improvement in Boon's NPH score by at least 15% or mRS by at least one point	For Rout ≥ 12 mmHg/ml/min, mRS ≥1 improvement Sensitivity: 85% Specificity: 21%
Takeuchi et al. (28); Japan	LIT protocol: a transducer was inserted for microsensor epidural pressure monitoring into the epidural cavity. The appearance of pathological pressure waves and basic epidural pressure was investigated by EDPM (5–9 h, mean 7.5 h) LP was performed in all patients during EDPM, and Rout was calculated from the infusion test using a 10–15 ml single bolus of saline injected at a rate of 1 ml per second **Criteria for positive LIT: Rout** **>** **20 mmHg/ml/min**	Time interval between LIT and shunt surgery: NR. Timing of outcome assessment: NR Shunt surgery was considered effective when the iNPH grade improved by one or more	Rout ≥ 20 Sensitivity 91.6% Specificity 92.3%
Sorteberg et al. (29); Norway	LIT protocol: the hydrodynamic assessment consisted of continuous monitoring of ICP for approximately 24 h, followed by a lumbar constant-rate infusion test. Intracranial pressure was monitored using a CodmanR pressure sensor. Under local anesthesia, the sensor was introduced 0.5–1 cm into the brain parenchyma via a small burr hole in the skull **Criteria for positive LIT: Rout exceeded 12 mmHg/ml/min**	Timing of shunt following index test: not reported Outcomes of the shunt were assessed approximately 6 months postoperatively. Criteria for shunt responsiveness: not mentioned	Sensitivity 86.7 Specificity 50
Eide and Sorteberg (30); Norway	LIT protocol: the lumbar puncture needle was connected to a transducer. The lumbar cerebrospinal fluid pressure (CSFP) was recorded continuously, both before and during infusion, with a Ringer solution at a standard infusion rate of 1.5 ml/min. The resistance to CSF outflow (Rout) was calculated by recording the opening pressure (Po) before infusion and the plateau (Pp) pressure obtained during infusion **Criteria for positive LIT: Rout** **>** **12 and Rout** **>** **18 were considered for the calculation of diagnostic accuracy measures** Those not shunted had a Rout < 15	The shunt was implanted 2–3 weeks after preoperative assessment. Timing of outcome assessment: 1 year after shunt Criteria for shunt responsiveness: Bech iNPH score improvement ≥3	Rout ≥ 12 Sensitivity 88% Specificity 45%
Hasselbalch et al. (31); Denmark	LIT protocol: investigations are performed using an automated CSF infusion method. CSF pressure is measured through two needles inserted into the CSF space at the L3–L4 interspace. One needle is used to infuse CSF, the other records pressure. Rout and the PA, as well as the PA/ICP ratio, were chosen as the main outcomes of the infusion test **Criteria for positive LIT: Rout of 11, 15.7, and 18.9 were used for the predictive power testing**, corresponding to the 25th, 50th, and 75th percentiles. No specific thresholds were mentioned for the positive LIT test for diagnostic accuracy assessments. However, it is mentioned that Rout was included in the decision to shunt. However, patients were not systematically excluded based on the results of the CSF infusion test—diagnostic process, where a low Rout does not preclude patients from undergoing surgery if clinical symptoms and radiological features point to iNPH	Timing of shunt surgery: NR Time to outcome assessment mean 7.4 (2–20 months) Changes in gait and continence scores were evaluated separately using the Hellström et al. scale. Criteria for shunt responsiveness: a positive response was defined as a summary shunt response score ≥1	Rout ≥ 11 Sensitivity 76% Specificity 22%
Wikkelsö et al. (17); 13 centers in 9 European countries	LIT protocol: a lumbar puncture was performed in the morning with a 0.7 mm needle, and the mean CSF baseline pressure was registered for 5 min, followed by a 12 ml CSF drainage (for CSF biomarker analyses) After the drainage, saline was infused until CSF pressure returned to baseline. After 5 min of rest, a lumbar infusion test was performed using a constant infusion or constant pressure method; Rout was calculated **Criteria for a positive LIT: Rout** **>** **12, 18, and 8 were used to calculate diagnostic accuracy measures**	The timing of surgery after the index tests was not mentioned Improvement in the Hellström iNPH Scale was defined as a 5-point increase or more, and in the mRS as a 1-point decrease 111 had index test and outcomes data at 1 year	Rout >12 Sensitivity 70% Specificity 35% Rout >18 Sensitivity: 31% Specificity 88%

Takeuchi et al. (28) employed epidural pressure monitoring with a 10–15 ml bolus saline injection to calculate Resistance to Outflow (*R*_out_). Sorteberg et al. (29) performed a prolonged (24-h) continuous intracranial pressure (ICP) monitoring, followed by a constant-rate infusion test. Eide et al. (30) used a standard infusion rate of 1.5 ml/min of Ringer's solution and calculated the *R*_out_ from the opening pressure and plateau pressure. Hasselbalch et al. used an automated CELDA System (Likvor AB, Umeå, Sweden) with two needles for infusion and pressure recording, focusing on Rout, Pulse Amplitude (PA), and the PA/ICP ratio. Wikkelsö et al. (17) performed baseline pressure measurements, CSF drainage, and saline infusion to return to baseline, followed by a constant infusion or constant pressure method for *R*_out_ calculation. *R*_out_ cut-offs selected also differed significantly. Boon et al. (27) reported the diagnostic accuracy measures at various cut-offs. Takeuchi et al. used >20 mmHg/ml/min (28); Sorteberg et al. (29) Eide et al. (30), and Wikkelsö et al. (17) used a cutoff >12 mmHg/ml/min. Wikkelsö et al. (17) also explored *R*_out_ >18 and >8. Hasselbalch et al. (31) used *R*_out_ percentiles (11, 15.7, 18.9) for predictive power testing without a single fixed threshold for positivity. The presence of pathological pressure waves (28) and other parameters, such as PA and the PA/ICP ratio (31), was also considered.

Eide et al. (30) performed shunt surgery 2–3 weeks after the LIT, and the other five studies did not report the time interval between the index test and the reference standard. As in studies on CSF-TT, the criteria for improvement following shunt surgery varied across five studies. Sorteberg et al. reported the number of patients who improved following surgery, according to criteria that were not explicitly stated (29). The timing of outcome assessments had similar shortcomings. We excluded Takeuchi et al. from the primary analysis since they used different diagnostic thresholds.

Five studies, including 368 patients (17, 27, 29–31), were included in the bivariate random-effects meta-analysis evaluating the diagnostic accuracy of the lumbar infusion test for predicting shunt responsiveness at a Rout cut-off of 11 (31) or 12 mmHg/ml/min (17, 27, 29, 30). The 2x2 contingency table for each included study is presented in Supplementary Table 4. The pooled sensitivity was 77.5% (95% *CI*, 70.2–83.4%) and the pooled specificity was 28.3% (95% *CI*, 19.0–39.9%; Figure 3), as also depicted by the hSROC in Supplementary Figure 2. Low between-study heterogeneity was observed for both sensitivity (τ^2^ = 0.05, *I*^2^ = 14.7%) and specificity (τ^2^ = 0.03, *I*^2^ = 4.6%). The generalized *I*^2^ statistic, reflecting overall heterogeneity across the joint bivariate distribution of sensitivity and specificity, was 0.00 with a tau^2^ of 0.00. The covariance between sensitivity and specificity was −0.04, with a correlation coefficient of −1.00. The bivariate model was fitted using adaptive Gauss–Hermite quadrature with five integration points. Non-concavity was observed at two of four iterations during the initial phase refining starting values, and briefly again at the first iteration of gradient-based optimization; the model then converged smoothly over the remaining iterations to a final log likelihood of −20.80, with no non-concavity at the final solution.

**Figure 3:**
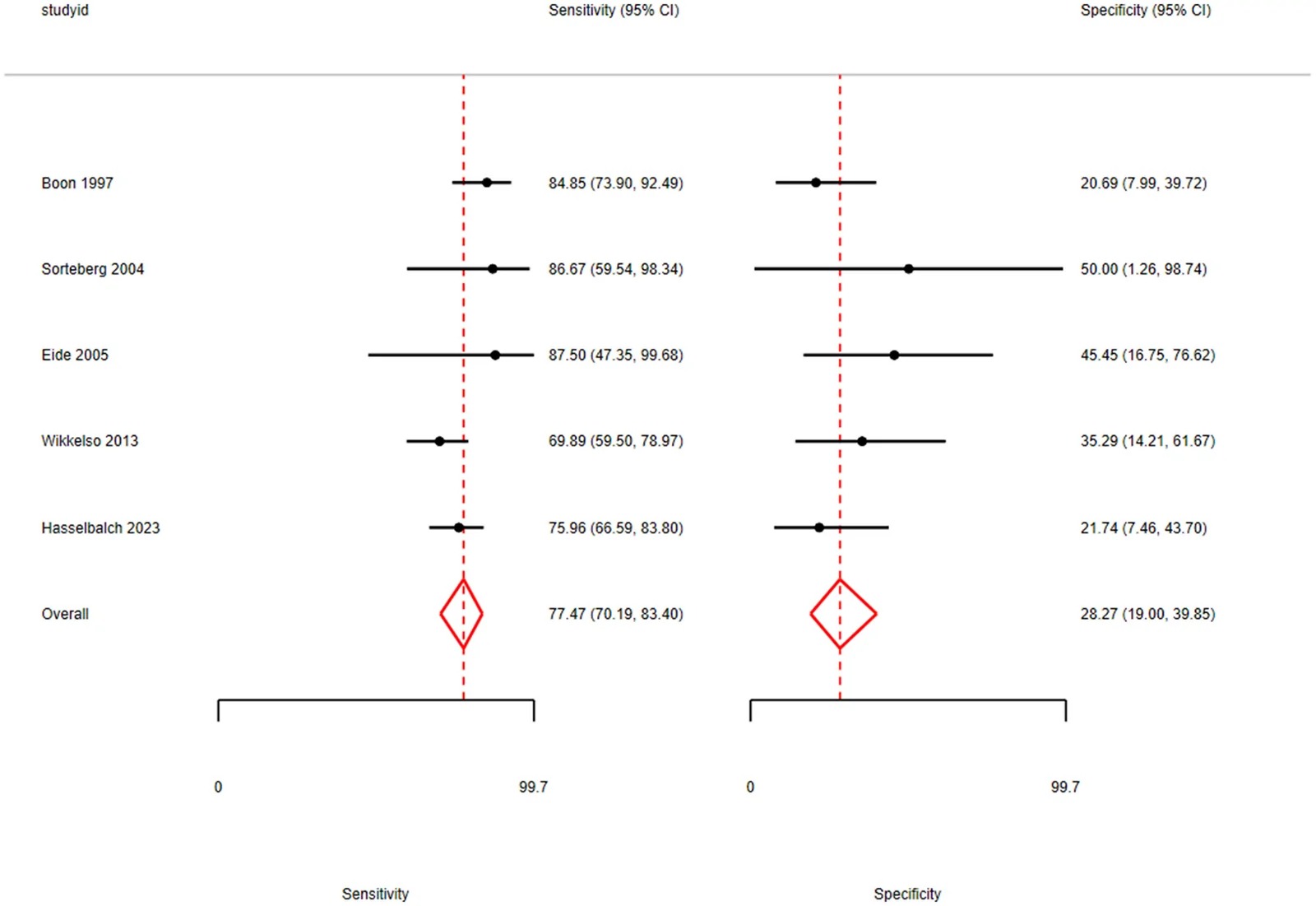
Forest plot showing the pooled sensitivity and specificity of the lumbar infusion test (LIT).

### Sensitivity analysis

A *post hoc* sensitivity analysis was performed for the CSF-TT by including Wikkelsö et al. (18), which enrolled patients with secondary NPH (post-subarachnoid hemorrhage, head trauma, and cerebrovascular disease) in addition to iNPH. Including this study, the ten-study pool (*n* = 721) yielded a pooled sensitivity of 66.2% (95% *CI*, 52–78%) with substantial between-study heterogeneity (τ^2^ = 0.76, *I*^2^ = 80%), and a pooled specificity of 54.5% (95% *CI*, 42.6–65.9%) as shown in Supplementary Figure 3, with moderate between-study heterogeneity (τ^2^ = 0.37, *I*^2^ = 47.3%). The generalized *I*^2^ statistic, reflecting overall heterogeneity across the joint bivariate distribution of sensitivity and specificity, was 0.00 with a tau^2^ of 0.04. The covariance between sensitivity and specificity was −0.53, with a correlation coefficient of −1.00. A leave-one-out analysis (Supplementary Table 5) of this ten-study pool, excluding each study individually, yielded pooled sensitivity estimates ranging from 60.99% to 70.10% and specificity estimates ranging from 51.62% to 59.00%, with the correlation coefficient remaining fixed at −1.00 in every iteration. The *post hoc* sensitivity analysis of the four studies (19–21, 23) that reported a 10% improvement in the TUG test response after the CSF-TT revealed a pooled sensitivity of 53.4% (95% *CI* 24.7–79.9%) and specificity of 61% (42–77.1%). The summary LR+ was 1.37 (95% *CI* 0.90–2.07), and the LR– was 0.76 (95% *CI* 0.46–1.26). The univariate analysis yielded a pooled sensitivity of 62.6% (95% *CI* 48.6–75.7%) and specificity of 52.3% (95% *CI* 40.7–63.9%).

For the LIT, a *post hoc* sensitivity analysis was performed, including Takeuchi et al. (28), which enrolled patients with atypical iNPH and used a different Rout cut-off (≥20 mmHg/ml/min) than the remaining studies (≥12 or ≥11 mmHg/ml/min). Including this study, the six-study pool (*n* = 393) yielded a pooled sensitivity of 80.9% (95% *CI*, 70.3–88.3%) with low between-study heterogeneity (τ^2^ = 0.13, *I*^2^ = 27.6%), and a pooled specificity of 42.8% (95% *CI*, 20.8–68.2%) as shown in Supplementary Figure 4 with substantial between-study heterogeneity (τ^2^ = 1.21, *I*^2^ = 60.8%). The generalized *I*^2^ statistic, reflecting overall heterogeneity across the joint bivariate distribution of sensitivity and specificity, was 35.6 with a tau^2^ of 0.08. The covariance between sensitivity and specificity was 0.28, with a correlation coefficient of 0.69, suggesting a positive relationship between the two measures; in contrast, the primary analysis indicated that the inclusion of Takeuchi et al. substantially influenced the between-study correlation and specificity estimates. Separately, a *post hoc* leave-one-out analysis of the primary five-study pool (Supplementary Table 6), excluding each study individually, yielded pooled sensitivity estimates ranging from 74.55% [excluding Boon et al. (27)] to 80.37% [excluding Wikkelsö et al. (17)] and specificity estimates ranging from 25.50% [excluding Eide and Sorteberg (30)] to 32.08% [excluding Boon et al. (27)], with the correlation coefficient at a boundary value in every iteration [−1.00 in four exclusions and +1.00 when Wikkelsö et al. (17) was excluded]. The leave-one-out analysis excluding Hasselbalch et al. (31) which used the Rout cut-off of 11 mmHg/ml/min, did not alter the pooled estimates. A univariate analysis of the five-study pool revealed a sensitivity of 78.6% (95% *CI* 71.7–84.8%) and a specificity of 25.9% (95% *CI* 15.7–37.3%).

### Likelihood ratios and post-test probability of shunt responsiveness

Based on the output of the bivariate modeling, the CSF-TT had an LR+ of 1.42 (95% *CI* 1.19–1.69) and an LR– of 0.66 (95% *CI* 0.51–0.84). Using the univariate sensitivity and specificity estimates of the CSF-TT under an independence assumption, LR+ was 1.31 (95% *CI* 0.87–1.75) and LR– was 0.72 (95% *CI* 0.40–1.03). A confirmatory analysis refitting the bivariate model with the correlation constrained to zero, fit directly to the study-level data, gave closely comparable results for the CSF-TT [LR+ = 1.33 (95% *CI* 0.89–1.77) and LR– = 0.70 (95% *CI* 0.39–1.01)].

The LIT had an LR+ of 1.08 (95% *CI* 0.93–1.26) and an LR– of 0.80 (95% *CI* 0.52–1.21). Using the univariate sensitivity and specificity estimates under an independence assumption, LR+ for the LIT was 1.06 (95% *CI* 0.88–1.24) and LR– was 0.83 (95% *CI* 0.39–1.26). Confirmatory analysis with the correlation constrained to zero gave sensitivity and specificity estimates similar to the primary model, with LR+ = 1.08 (95% *CI* 0.91–1.26) and LR– = 0.79 (95% *CI* 0.43–1.16).

As shown in Table 3, post-test probabilities of shunt responsiveness across a range of assumed pre-test probabilities did not produce clinically meaningful shifts for either test: a positive result raised the probability of shunt responsiveness only modestly, while a negative result lowered it only slightly, regardless of whether the pre-test probability was assumed to be low, moderate, or high.

**Table 3:** Post-test probability of shunt responsiveness with a positive or negative result of CSF-TT and LIT across various pre-test probability thresholds.

Pre-test probability	Post-test probability of shunt responsiveness with a positive result	Post-test probability of shunt responsiveness with a negative result	Interpretation
CSF tap test [sensitivity 64.1% (95% *CI* 49.2–76.6%), specificity 54.8% (95% *CI* 41.8–67.2%)]			
The test has limited ability to meaningfully rule in or rule out shunt responsiveness			
Bivariate LR+ = 1.42 (95% *CI* 1.19–1.69) Univariate LR+ = 1.31 (95% *CI* 0.87–1.75)		A positive CSF-TT does not reliably increase the likelihood of shunt responsiveness: the sensitivity-analysis confidence interval for LR+ crossed 1, and the estimate is unreliable due to the boundary correlation. Primary model results should be interpreted with caution	
Bivariate LR– = 0.66 (95% *CI* 0.51–0.84) Univariate LR– = 0.72 (95% CI 0.40–1.03)		A negative CSF-TT does not reliably decrease the likelihood of shunt responsiveness: the sensitivity-analysis confidence interval for LR**–** crossed 1, and the estimate is unreliable due to the boundary correlation. Primary model results should be interpreted with caution	
50%	59% (95% *CI*: 46% to 70%)	40% (95% *CI*: 26% to 55%)	At a pre-test probability of 50%, 59% of people with a positive test result and 40% of people with a negative test result will be shunt responsive
60%	68% (95% *CI*: 56% to 78%)	50% (95% *CI*: 34% to 65%)	At a pre-test probability of 60%, 68% of people with a positive test result and 50% of people with a negative test result will be shunt responsive
80%	85% (95% *CI*: 77% to 90%)	72% (95% *CI*: 58% to 83%)	At a pre-test probability of 80%, 85% of people with a positive test result and 72% of people with a negative test result will be shunt responsive
Lumbar infusion test [sensitivity 77.5% (95% CI 70.2–83.4%), specificity 28.3% (95% CI 70.2–83.4%)]			
The test has minimal value in distinguishing shunt responders from non-responders			
Bivariate LR+= 1.08 (95% *CI* 0.93–1.26) Univariate LR+ = 1.06 (95% *CI* 0.88–1.24)		A positive LIT does not reliably increase the likelihood of shunt responsiveness; the confidence intervals for LR+ crossed 1 in the primary model as well as the sensitivity analysis	
Bivariate LR– = 0.80 (95% *CI* 0.52-−1.21) Univariate LR– = 0.83 (95% *CI* 0.39–1.26)		A negative LIT does not reliably decrease the likelihood of shunt responsiveness; the confidence intervals for LR– crossed 1 in the primary model as well as the sensitivity analysis	
50%	52% (95% *CI*: 46% to 58%)	45% (95% *CI*: 30% to 61%)	At a pre-test probability of 50%, 52% of people with a positive test result and 45% of people with a negative test result will be shunt responsive
60%	62% (95% *CI*: 56% to 67%)	55% (95% *CI*: 39% to 70%)	At a pre-test probability of 60%, 62% of people with a positive test result and 55% of people with a negative test result will be shunt responsive
80%	81% (95% *CI*: 78% to 85%)	77% (95% *CI*: 63% to 86%)	At a pre-test probability of 80%, 81% of people with a positive test result and 77% of people with a negative test result will be shunt responsive

LR+ positive likelihood ratio; LR– negative likelihood ratio.

LR+ and LR– are conventionally interpreted as follows (36): LR+ greater than 10 or LR– less than 0.1, large and often conclusive shift in probability; LR+ 5–10 or LR– 0.1–0.2, moderate shift; LR+ 2–5 or LR– 0.2–0.5, small but sometimes important shift; LR+ 1–2 or LR– 0.5–1, small, rarely important shift; LR+ or LR– equal to 1, no change in probability. A test is generally considered clinically useful only when LR+ exceeds 2 or LR– falls below 0.5.

Post-test probabilities for positive and negative index test results were calculated by applying pooled sensitivity and specificity to pre-test probabilities of 50%, 60%, and 80%, with confidence intervals derived by substituting the corresponding sensitivity and specificity confidence limits.

### Risk of bias

The studies demonstrated various sources of bias, particularly in patient selection, the conduct and reporting of the index tests and reference standards, and patient flow, as detailed in Table 4, which lists the per-study responses to each QUADAS-2 signaling question across all four domains, and in Supplementary Table 7. Although none of the studies employed a case-control methodology, and most used consecutive patient series, eight studies had a high risk of bias, and one had an unclear risk of bias due to inappropriate patient exclusion, raising concerns about the applicability of the results. Applicability concerns were also identified in several studies, notably Takeuchi et al. (28), which included only patients with atypical iNPH; Wikkelsö et al. (18) included patients with secondary NPH (post-subarachnoid hemorrhage, head trauma), and Hasselbalch et al. (31) had unclear applicability concerns because it was unclear if all clinico-radiologically diagnosed patients were selected for study enrollment.

**Table 4:**
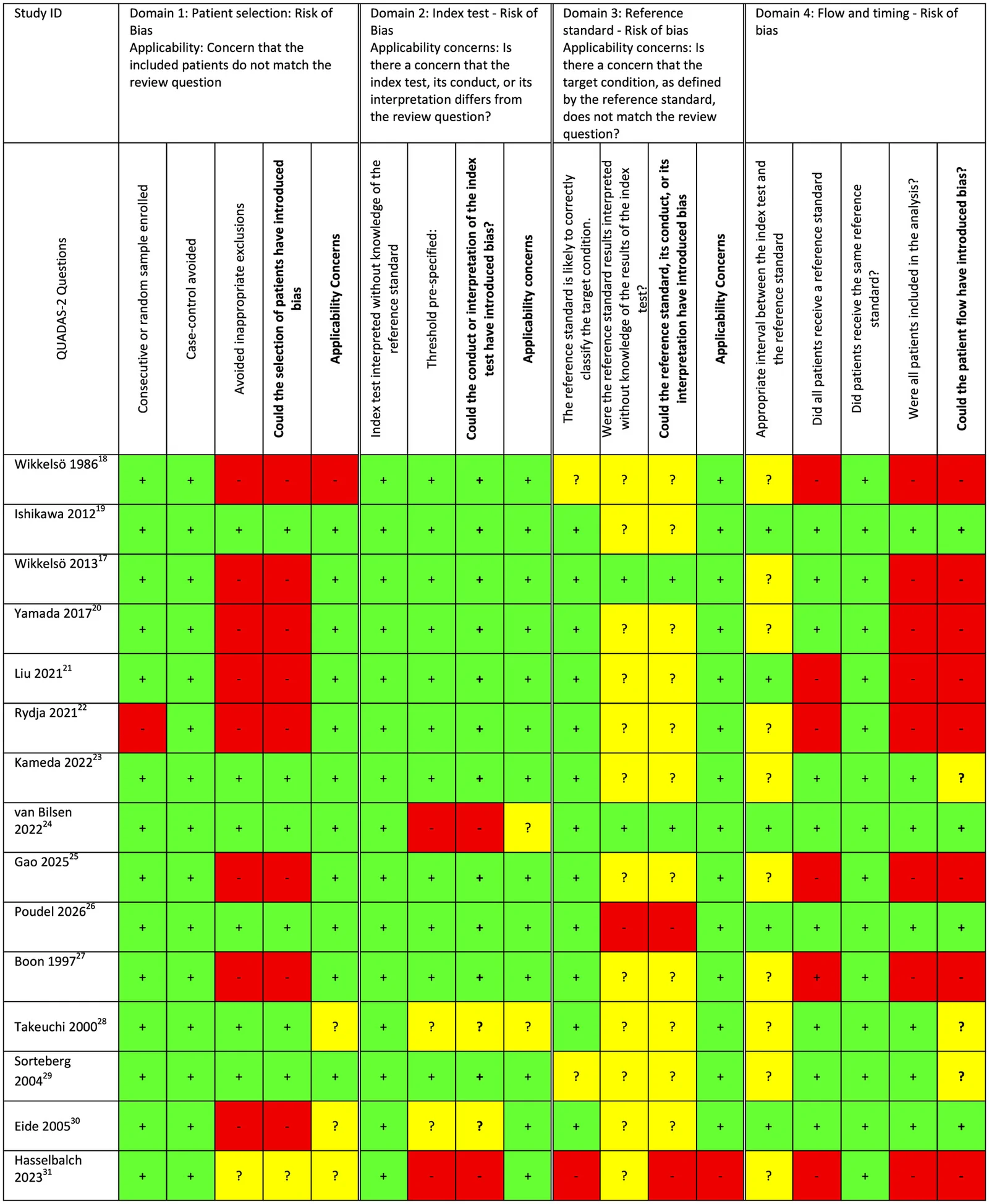
Risk of bias summary: QUADAS-2.

Colour coding for Risk of bias Red-High risk, Yellow: Unclear risk; Green low risk.

With the index tests, namely CSF-TT and LIT, most studies were reported without knowledge of the reference standard; however, some studies did not report or did not have prespecified thresholds, leading to unknown or high risk of bias. For the reference standard, some studies did not report the criteria for assessing improvement following shunt surgery, making it difficult to determine whether the target condition, in this case, iNPH, was correctly identified. Most studies did not report whether the reference standard was interpreted without knowledge of the index test, making the risk of bias unclear. In Poudel et al. (26), the reference standard assessor had access to the index test results, which put it at high risk of bias.

In the domain of flow and timing, only Eide and Sorteberg (30), Ishikawa et al. (19), van Bilsen et al. (24), and Poudel et al. (26) met the criteria for low risk of bias. Eight studies had a high risk of bias as they did not include all the patients in the analysis (Table 4).

### Summary of certainty of evidence

#### CSF tap test

The certainty of the evidence was very low for sensitivity, and it was downgraded for risk of bias, inconsistency, and imprecision. Based on low-certainty evidence, downgraded for bias and inconsistency, the entire 95% confidence interval of the pooled specificity fell below the 70% threshold for predicting response to shunt surgery, suggesting a considerable false-positive rate (Supplementary Table 8). The pooled sensitivity was 64.1% (95% *CI*: 49.2–76.6%); the lower bound of this interval (49.2%) fell below even a more permissive 50% threshold, confirming that serious imprecision persisted regardless of whether a 70% or 50% threshold was applied.

#### Lumbar infusion test

There is moderate-certainty evidence for the LIT's sensitivity in predicting response to shunt surgery, downgraded due to serious risk of bias. Additionally, there is moderate-certainty evidence, downgraded for bias, that the LIT shows a poor specificity (95% *CI* entirely below 70%) for predicting response to shunt surgery, suggesting that a considerable proportion of non-responders may be incorrectly classified as likely responders (Supplementary Table 9).

## Discussion

Both the CSF-TT and LIT are widely used diagnostic tools in the evaluation of iNPH. However, there is significant heterogeneity in the protocols for performing these tests, in the timing of assessment following the CSF-TT, the type of gait, the urinary or cognitive assessment tools used, the cut-offs, and the criteria used to assess CSF-TT responsiveness or LIT positivity, as identified by our systematic review. Similarly, we found significant differences in the timing of shunt surgery after the index test, the timing of postoperative outcome following the shunt surgery, and the criteria used to define shunt responsiveness. Several studies made no explicit mention of these case definitions, making it difficult, if not impossible, to determine how the diagnosis was established.

The diagnosis of “definite” NPH is made based on response to shunt surgery (4). This is somewhat counterintuitive, as the diagnosis, according to this definition, would be made after the treatment. In medicine, we prefer to establish a diagnosis before offering an invasive surgical procedure. However, the lack of standardization of diagnostic tests currently impedes the assessment of diagnostic accuracy in NPH.

CSF-TT is generally considered less invasive and simpler to perform. It also gives clinicians a sense that, since it mimics CSF drainage by shunt surgery, it intuitively makes sense that only patients with a positive tap test should undergo definitive surgery, thereby making it a common initial screening tool for referring patients for surgical treatment. However, the currently available literature does not support this notion. The guidelines (4), therefore, state that, regardless of the tap test results, patients may be referred for surgery, as is the practice in Japan. In line with these recommendations, we found that the sensitivity of the CSF-TT was 64.1% (95% *CI*: 49.2–76.6%), meaning that several patients who were CSF-TT non-responders still responded to VP shunting.

In contrast, although the LIT is a more invasive procedure, it is considered to provide quantitative physiological data on CSF dynamics, such as *R*_out_ and ICP PA. While some studies report exceptionally high accuracy (e.g., Takeuchi et al. (28) reported 91.6% sensitivity and 92.3% specificity using a *R*_out_ cut-off ≥20 mmHg/ml/min), others show concerningly low specificity (e.g., 22% specificity with a cut-off ≥11 in Hasselbalch et al. (31)), indicating a potential risk of false positives. The lack of consensus on pathological *R*_out_ values and the influence of methodological variations on measured parameters remain significant limitations. These variations in infusion methods, pressure-monitoring techniques, and the specific parameters measured make it difficult to compare these studies consistently.

Both the CSF-TT and LIT were evaluated using the GRADE approach to assess the certainty of evidence for diagnostic accuracy in predicting response to shunt surgery in patients with iNPH. The CSF-TT demonstrated a sensitivity of 64.1% (95% *CI*: 49.2–76.6%); however, the certainty of evidence was rated very low. This was due to a serious risk of bias in the included studies, a very serious inconsistency arising from statistical heterogeneity (evidenced by a high τ^2^ and *I*^2^), and serious imprecision, as the lower bound of the confidence interval crossed the threshold of diagnostic utility of 70% or even a more permissive threshold of 50%. In contrast, there is low-certainty evidence that the specificity of CSF-TT was poor at 54.8% (95% *CI*, 41.8–67.2%), due to a serious risk of bias and serious inconsistency.

Similarly, the LIT showed a pooled sensitivity of 77.5% (95% *CI*: 70.2–83.4). The certainty of evidence was rated as moderate, downgraded due to a serious risk of bias resulting from methodological limitations across the studies. The specificity of LIT was poor at 28.3% (95% CI: 19.0–39.9), with moderate certainty of evidence for poor specificity, downgraded due to a serious risk of bias. The 95% confidence interval for sensitivity remained entirely above both the 70% threshold and a more permissive 50% threshold. As regards specificity, again, the entire 95% confidence interval was below the threshold of 70%, indicating LIT's poor specificity. It should also be noted that Wikkelsö et al. (17) contributed data to both the CSF-TT and LIT pooled analyses, as this cohort underwent both index tests; while this reflects real-world co-administration of the two tests, it introduces a degree of non-independence between the two meta-analyses that should be considered when comparing the CSF-TT and LIT estimates directly.

An earlier review by Mihalj et al. (7) reported high specificity and positive predictive value for predicting shunt responsiveness; however, this pooled estimate combined studies of the CSF-TT with studies employing external lumbar drainage (ELD), a more invasive test involving continuous CSF drainage, which likely inflated the apparent specificity relative to the CSF-TT alone. Our review, in contrast, exclusively evaluated the CSF-TT, including the recently published studies, the test used far more widely in routine clinical practice, rather than pooling it with ELD, and found a substantially lower pooled specificity, a result we consider more representative of the CSF-TT's real-world diagnostic performance. A recent meta-analysis by van Bilsen et al. (32) similarly reported poor LIT specificity across Rout cut-offs, with very low negative predictive value. The present review extends these findings by providing a joint bivariate analysis of both CSF-TT and LIT, applying formal GRADE certainty ratings, and incorporating explicit criteria to avoid circular reasoning in study inclusion.

Sensitivity analyses did not change the GRADE certainty ratings for the CSF-TT estimates. This reflects the stability of the categorical GRADE judgment rather than validation of the underlying bivariate model. For the LIT, inclusion of Takeuchi et al. (28) yielded a notably higher point estimate of the specificity, underscoring the influence of patient population and Rout threshold on pooled estimates, though GRADE ratings remained unchanged.

These findings underscore that while CSF-TT and LIT may help identify patients who could benefit from shunt surgery, their limited specificity and very low certainty of evidence caution against their use as standalone diagnostic tools; when interpreted alongside clinical, radiological, and physiological findings as part of a multimodal assessment, they may still contribute to the diagnostic pathway. This is reinforced by the modest likelihood ratios for both tests (LR+ 1.42 and LR– 0.66 for the CSF-TT; LR+ 1.08 and LR– 0.80 for the LIT), which fall short of the conventional threshold for clinical usefulness and translate into only marginal shifts in the post-test probability of shunt responsiveness regardless of a positive or negative result. There remains a critical need for higher-quality studies with standardized protocols and blinded assessments to better define their diagnostic value. The inconsistent performance of both tests, particularly when using clinical outcome measures or fixed cut-offs, strongly suggests that NPH shunt responsiveness is a complex, multifactorial phenomenon unlikely to be captured by a single, simple test. If multiple underlying pathophysiological mechanisms contribute to a complex medical condition, it is highly unlikely that a single diagnostic marker will be perfectly predictive of treatment response. This reinforces the need for a standardized multimodal assessment approach that integrates various clinical, radiological, and physiological parameters.

Our review has a few limitations that warrant consideration. We excluded studies that were not in English, which could have introduced selection bias. ELD (external lumbar drainage) was not included in our search strategy or eligibility criteria; the review protocol was registered, and a systematic search strategy was created. ELD was not in the strategy, and studies were therefore not systematically searched or screened for inclusion. Nonetheless, ELD is regarded by some authors as an accurate predictor of shunt responsiveness, with a systematic review by Nunn et al. (33) reporting pooled sensitivity of 94% (95% *CI* 41–100%) and specificity of 85% (95% *CI* 33–100%) across four small studies. Clinicians in centers where ELD is feasible may wish to consider it alongside or instead of CSF-TT/LIT, particularly given the modest specificity we found for both tests; however, ELD was beyond the scope of the current review. Many of the included studies were retrospective in design and involved heterogeneous patient populations.

In some cases, outcome reporting was incomplete, and patients with surgical complications, such as postoperative bleeding, were excluded from final analyses, potentially influencing the interpretation of results. Additionally, there was variation in the protocols used for index tests and outcome assessment, reflecting the lack of standardized approaches at the time. Several of these studies were conducted decades ago, when reporting standards were still evolving, which is reflected in their overall methodological quality. As a result, concerns regarding the risk of bias and applicability were raised in several studies. Therefore, the findings on diagnostic accuracy should be interpreted with these limitations in mind. We did not perform a sensitivity analysis restricted to studies with low risk of bias, as none of the included studies had low risk of bias across all four QUADAS-2 domains, rendering such an analysis impossible. Heterogeneity of studies is another major issue. Pooled estimates are derived from highly heterogeneous protocols. Thresholds for test positivity, timing of outcome assessment, and definition of “shunt response” varied significantly, limiting the external validity and generalizability of the results. We did not assess for publication bias, and funnel plots were not generated due to the limited number of studies per test; therefore, small-study effects could not be ruled out.

The correlation coefficient of −1.00 obtained for both the CSF-TT and LIT primary bivariate analyses is a boundary estimate: estimation converged at the edge of the permissible parameter space (±1) rather than at an interior value, indicating that the between-study covariance parameter could not be reliably estimated from the available data. It should not be interpreted as evidence of a true, biologically meaningful inverse relationship between sensitivity and specificity. Along the same vein, the likelihood ratio calculations of the CSF-TT were not robust to the boundary correlation estimate, as the primary model's intervals excluded 1, whereas both sensitivity analyses produced wider intervals that included 1 (Table 3). The leave-one-out sensitivity analysis showed that the correlation coefficient remained fixed at −1.00 regardless of which study was excluded, indicating that this is a structural feature of the bivariate model given the limited number of studies rather than an artifact driven by any single study; the consistently narrow ranges observed for pooled sensitivity and specificity across all exclusions indicate that these point estimates were relatively stable to the exclusion of any single study. A parallel leave-one-out analysis of the primary five-study LIT pool showed the same pattern: the correlation coefficient sat at a boundary value in every exclusion, whereas pooled sensitivity and specificity remained narrowly consistent across all exclusions; this confirms that, as with the CSF-TT, the correlation and heterogeneity parameters for the LIT model are not reliably estimable at this sample size; the headline accuracy point estimates were relatively stable across exclusions. As a further check, a univariate random-effects model fitted separately for sensitivity and specificity of CSF-TT and LIT both yielded closely comparable pooled estimates, supporting that the bivariate model's accuracy estimates were not an artifact of the boundary correlation. Because of these issues with unestimable correlation, the likelihood ratios should be interpreted with caution.

Since the currently available ancillary tests are not sufficiently accurate, we need better prediction models of shunt responsiveness based on clinical features, imaging (16), CSF studies, and biomarkers (34). A recent study described the potential role of CSF biomarkers QPCT and RBP4 identified based on machine learning in a small cohort of 37 patients, which was validated in an independent 12-patient cohort (35). We need multimodal predictors that are developed and validated in large multicentre cohorts to help patients and clinicians make informed choices.

## Conclusion

This systematic review found that both CSF-TT and LIT have limited diagnostic accuracy in predicting shunt responsiveness in iNPH. The pooled specificity of CSF-TT was 54.8% (95% *CI* 41.8–67.2%, low certainty evidence) and that of LIT was 28.3% (95% *CI* 19.0–39.9%, moderate certainty evidence), indicating poor specificity for both tests.

In practical terms, the poor specificity of the CSF-TT (54.8%) means that a substantial proportion of patients who do not respond to shunt surgery will still test positive on the tap test (a false-positive rate of 45.2%), which risks recommending surgery to non-responders; the proportion of positive results that represent true responders, the positive predictive value depends on the prevalence of shunt responsiveness in the population being tested and cannot be inferred from specificity alone. Similarly, a positive LIT result should be interpreted with caution, given its poor specificity (28.3%), supported by moderate-certainty evidence downgraded for serious risk of bias.

Given these findings, clinicians should exercise caution when relying on these tests in isolation to guide surgical decisions. However, they may still contribute useful information within a broader, multimodal diagnostic assessment. Conversely, the sensitivity of the CSF-TT (64.1%) means that approximately one in three true shunt responders will test negative, potentially denying surgery to patients who would benefit. These limitations are even more pronounced for the LIT, where a specificity of 28.3% means that nearly three-quarters of non-responders would test positive. Test results should therefore not be interpreted in isolation, and management decisions should incorporate the full clinical picture, imaging findings, and patients' values and preferences. Prospective studies using standardized protocols with clearly defined outcome parameters following shunt surgery are needed.

Moving forward, the immediate priority should be standardizing patient selection criteria, assessment tools, and outcome definitions across centers. Studies should require documented gait dysfunction as an inclusion criterion, and standardized gait assessments, such as the 10-meter walk test, should be uniformly adopted. In the near term, well-designed prospective trials in which all patients undergo shunt surgery regardless of index test result are needed to establish true diagnostic accuracy and break the circularity. Finally, future efforts should explore multimodal predictive models integrating clinical, imaging, and biomarker data, which may ultimately yield more accurate, individualized predictions of shunt responsiveness than any single test.

## Data Availability

The original contributions presented in the study are included in the article/Supplementary material, further inquiries can be directed to the corresponding author.

## Supplementary material

Supplementary file 1 PRISMA-DTA checklist—preferred reporting items for systematic review and meta-analysis of diagnostic test accuracy studies.

Supplementary file 2 Search strategy.

Supplementary Figure 1 Hierarchical Summary receiver operating characteristic (hSROC) curve for CSF-TT.

Supplementary Figure 2 Hierarchical Summary receiver operating characteristic (hSROC) curve for LIT.

Supplementary Figure 3 Forest plot showing pooled sensitivity and specificity of the CSF-TT *post-hoc* sensitivity analysis, including Wikkelsö et al. (18) (ten-study pool).

Supplementary Figure 4 Forest plot showing pooled sensitivity and specificity of the LIT *post-hoc* sensitivity analysis, including Takeuchi et al. (28) (six-study pool).

Supplementary Table 1 Demographics and Clinical Details of included studies reporting the diagnostic accuracy of the CSF-TT.

Supplementary Table 2 CSF Tap Test Diagnostic 2 × 2 Data for the included studies.

Supplementary Table 3 Demographics and Clinical Details of included studies reporting the diagnostic accuracy of the LIT.

Supplementary Table 4 Lumbar Infusion Test (LIT) Diagnostic 2 × 2 Data of the included studies.

Supplementary Table 5 Leave-one-out sensitivity analysis for CSF-TT.

Supplementary Table 6 Leave-one-out sensitivity analysis of LIT.

Supplementary Table 7 Risk of bias assessment QUADAS 2.

Supplementary Table 8 GRADE summary of findings table for the CSF-TT.

Supplementary Table 9 GRADE summary of findings table for the LIT.
