# Supplementary material for: Accuracy of CSF tap test and lumbar infusion test in predicting shunt response in idiopathic normal pressure hydrocephalus: a systematic review and meta-analysis
Source: Front Neurol. 2026 Oct 12;17:1908328. doi: 10.3389/fneur.2026.1908328 (PMC13710815; doi:10.3389/fneur.2026.1908328)
Supplement: Supplementary file 1 [file Data_Sheet_1.pdf]

Supplementary Table 1: Demographics and Clinical Details of included studies reporting the diagnostic accuracy of the CSF-TT

**Study ID; Country; Duration**<br>**Aims, Population description, Inclusion and exclusion criteria**	**Mean age, Clinical profile, Evans index, and baseline scores**
1. **Wikkelsö 1986**;[1] Sweden; 1979-1983; Diagnostic test accuracy study<br>Funding: grants from the Edit Jacobson's Foundation, the Medical Faculty of Gothenburg, the Medical Society of Gothenburg, and the Swedish Medical Research Council<br>Aim of the study: Evaluation of the predictive value of the CSF-TT<br>Participants: Patients who were investigated for NPH and underwent VP shunt<br>Inclusions: consecutive patients evaluated and operated with a VP shunt; only 6 had iNPH, 9 had SAH, 8 had head trauma, and 4 had cerebrovascular diseases<br>Selection for shunt surgery was based on characteristic changes observed during clinical investigation, CT, and radionuclide cisternography. Inflammatory and metabolic disorders were excluded by appropriate CSF and blood examinations; 3 patients with complications were excluded from the analysis.<br>**CSF Tap test was not used in the decision-making process for shunting**	**27 patients**; Mean age: 63 (40-81); M/F: 13/14;<br>Vascular disease: NR; Cognitive impairment: NR<br>Gait dysfunction: 89%, Urinary dysfunction: 81%; Duration of symptoms: 21 months (3 months – 9 years)<br>Evans Index: 0.38 (0.30-0.46)<br>Baseline Gait score, cognitive and urinary scores: NR<br>CSF opening pressure: 12±5mm Hg<br>**Index test and outcome data available in 21 patients**
2. **Ishikawa 2012**;[2] Japan; 2004-2006; Prospective cohort study<br>Funding: Supported in part by Johnson and Johnson, Nihon Medi Physics, and Daichi Pharmaceuticals, honoraria from companies that manufactured devices;<br>Aim of the study: The predictive value of TT was investigated in patients with iNPH; validate the diagnostic importance of high-convexity tightness in coronal-section MRI with the results of shunt surgery using a programmable valve<br>Participants: Patients with iNPH who underwent VP shunt<br>Inclusions: (1) 60 to 85 years old, (2) one or more of the NPH triad symptoms, (3) Ventriculomegaly (Evans Index > 0.3), (4) high-convexity tightness in coronal-section MRI, and (5) no antecedent disorders.<br>**CSF Tap test was not used in the decision-making process for shunting**	**100 patients**; 80 shunt responders, 20 non-responders; Median age: 75; M/F: 58/42<br>Vascular disease: na; Cognitive impairment: 80%; Gait dysfunction: 91%; Urinary dysfunction: 60%<br>Duration of symptoms: NR; Evans Index: 34.6 (32.7-38); Baseline iNPH Gait score: 2 (2-3); Baseline iNPH Cognitive score: 2 (1-3); Baseline urinary score: 2 (1-3.7); Mean volume of CSF tapped: 30 mL; CSF opening pressure: 12(9-14) cm H20<br>**Index test and outcome data available in 100 patients**
3. **Wikkelsö 2013**;[3] 13 centers in 9 European countries; Sep 2004-Jan 2008; Prospective cohort study<br>Funding: Codman & Shurtleff, Raynham, Massachusetts, USA<br>Aim of the study: To determine the sensitivity, specificity, and positive and negative predictive values of the CSF TT and Rout in patients clinically presenting with iNPH.<br>Participants: iNPH; Inclusions: Gradually developed gait disturbance affecting tandem walking, turning, stride length, and width. The MRI criteria for iNPH were a symmetrical quadri-ventricular enlargement without cortical infarcts or other clinically relevant parenchymal lesions (lacunar infarcts of less than 1 mL were accepted), whereas single cortical infarcts were accepted for a diagnosis of iNPHQ. Free communication between the ventricular system and the subarachnoid space and an Evans Index greater than 0.30 were mandatory for both diagnoses.<br>Exclusions: Patients who were unwilling to participate, had a restricted life expectancy, or had contraindications to surgery<br>**CSF Tap test was not used in the decision-making process for shunting**	**115 patients**, Mean age: 71(9); M/F: 58/57; Vascular disease: 8%; Cognitive impairment, Urinary and gait dysfunction: Not reported<br>Duration of symptoms: 18 months (12-30)<br>Evans Index: 0.42 (0.31-0.66); Baseline Gait score, cognitive and urinary scores: NR<br>Mean volume of CSF tapped: 50ml; CSF opening pressure: 10 mmHg(2-18)<br>**111 had index test and outcome data at 1 year**
4. **Yamada 2017**;[4] Japan; March 2010-Oct 2011; Cohort study<br>Funding: The funding sources (Johnson & Johnson K.K. and NihonMedi-Physics Co. Ltd.)<br>Aim of the study: To investigate time duration since the initial presentation of iNPH symptoms until the CSF tap test as a pivotal factor that could increase the accuracy of predicting the iNPH diagnosis using the CSF tap test.<br>Participants: Possible iNPH according to the second edition of the Japanese iNPH guideline<br>Inclusions: Age at entry 60-85 years; patients with one or more of gait disturbance, cognitive impairment, urinary disturbance on the iNPH grading scale (iNPHGS) within 3 mo before the date of consent; ventriculomegaly with Evans index >0.3, in concurrent with narrow sulci at the high convexity and enlarged Sylvian fissure on CT or MRI.<br>Exclusions: Those with a diagnosis of secondary hydrocephalus that occurred after subarachnoid hemorrhage, meningitis, head trauma, congenital hydrocephalus, or aqueductal stenosis, among others; CSF pressure of ≥20 cm H2O; complications of severe disuse muscle atrophy; psychiatric disorders or other neurological diseases<br>**CSF Tap test was not used in the decision-making process for shunting**	**93 patients**; Mean age (SD) 76.5(4.5)<br>Vascular disease: NR; cognitive impairment, Gait dysfunction, urinary dysfunction: NR<br>Duration of symptoms: 24.2(1-120) mo Evans Index: NR<br>Baseline iNPH Gait score 2.3-2.5(0.7), iNPH Cognitive score 2.3-2.4 (0.9), and urinary score 2 (0.9), iNPH total score 6.6 (2.2)<br>Mean volume of CSF tapped, CSF opening pressure: NR<br>**82 patients had index tests and reference standard outcomes available.**
5. **Liu 2021**;[5] China; 2012-2019; Retrospective cohort study<br>Funding: Grant from National Key Research and Development Program of China, CAMS Innovation Fund for Medical Sciences (2016-12M-1-004), National Natural Science Foundation of China, and the Strategic Priority Research Program Biological basis of aging and therapeutic strategies of the Chinese Academy of Sciences<br>Aim of the study: To compare the improvement in TUG, 10 m walking time, 10 m walking steps, and composite Z score at 8 h, 24 h, and 72 h after CSF TT and calculate the specificity, sensitivity, PPV, and NPV of the walking tests and executive composite Z score according to the results of shunting follow-up assessments<br>Participants: clinically possible normal-pressure hydrocephalus according to international diagnostic criteria and Chinese consensus<br>Inclusions: 1. diagnosis with iNPH according to international guidelines and Chinese consensus; 2. completion of a multi-time point assessment of walking ability and neuropsychological status. Exclusions: The patients could not tolerate 30 mL of CSF drainage during the CSF TT<br>**CSF Tap test was not used in the decision-making process for shunting**	**88 patients**; Age 67±12 y<br>M/F: 61/27; Vascular disease: NR<br>Cognitive impairment: 71.6%; Gait impairment: 84%; urinary dysfunction: 67%<br>Duration of symptoms: 0.5-11 years<br>Evans Index, Baseline Gait, urinary, and cognitive scores: NR<br>**Index test and outcomes of reference standards available in 23 patients**

6. **Rydja 2021**;[6] Sweden; Jan 2016-Dec 2019; Retrospective cohort study<br>Funding: Open access funding provided by Linköping University<br>Aim of the study: To evaluate the prognostic value of the CSF TT using the Hellström iNPH scale among shunted iNPH patients in a large single-center cohort<br>Participants: Patients with a diagnosis of possible and probable iNPH, based on the international guidelines of 2005, and treated with an adjustable valve shunt<br>Inclusions: All patients had a disturbed gait and balance and, additionally, an impairment of cognition and/or continence symptoms. Exclusions: Patients were excluded if they had missing data for the CSF TT or the postoperative assessment.<br>**CSF Tap test was not used in the decision-making process for shunting**	**159 Patients**; Age: 75.4 (6.2); M/F: 62/54;<br>Vascular disease: 9.5%; Cognitive impairment, Gait dysfunction, and urinary dysfunction: NR<br>Duration of symptoms, Evans Index, Baseline Gait, and urinary scores: NR<br>Baseline Cognitive score: MMSE 26 (23-28), n=115; Total iNPH score 51.8(16.9)<br>Mean volume of CSF tapped: 48.0 (44.0-50.0)<br>CSF opening pressure: 15 (12.5-18.0) cm H20<br>**Index test and outcomes of the reference standard available in 116 patients.**
7. **Kameda 2022**;[7] Japan; Apr 2021-Sep 2021; Retrospective cohort study<br>Funding: Japan Society for the Promotion of Science Grant-in-Aid for Scientific Research;<br>Aim of the study: To study if the comprehensive evaluation of the effect of the tap test using the Functional Gait Assessment (FGA) and Global Rating of Change (GRC) scales can lead to more appropriate patient selection for shunting than the evaluation of the effects of TUG alone<br>Participants: Patients with a positive tap test who showed some improvement in any of the three symptoms: gait, urinary incontinence, and cognitive function after the tap test were determined to be eligible for LP shunt. In addition, patients with a negative tap test but DESH findings were identified as having a high probability of iNPH and were considered eligible for LP shunt.<br>**CSF Tap test was not used in the decision-making process for shunting**	**40 patients;** Mean age: 78.8(5.2)<br>M/F: 19/21<br>Vascular disease, Cognitive impairment, Gait dysfunction, urinary dysfunction: NR<br>Duration of symptoms, Evans Index: NR<br>Baseline TUG 15.3 (5.7); MMSE 24.5 (4.6); iNPH 5.2(2.4)<br>**TUG test outcomes available in 40; FGA in 24 patients**
8. **vanBilsen 2022**;[8] Netherlands; Jan 2015-Jan 2020; Prospective cohort study<br>Funding: Not reported<br>Aim of the study: A high delta amplitude would be a good diagnostic marker for a positive shunt response, and a low amplitude or inability to lower the amplitude would correlate with a negative shunt response. Combining delta amplitude with other diagnostic markers deduced from LIT, such as P0, Rout, and tap test, could improve specificity and sensitivity.<br>Participants: iNPH; Inclusions: Patients who underwent shunt surgery preceded by a LIT according to the new pulsatility curve protocol for diagnosis of iNPH<br>Exclusions: secondary NPH or aqueductal stenosis<br>**CSF Tap test was not used in the decision-making process for shunting**	38 patients; Mean age: 72; M/F: 24/14;<br>Vascular disease: 37%; Cognitive impairment: 32(84.2); Gait dysfunction: 100%, urinary dysfunction: 82%<br>Duration of symptoms, Evans Index, Baseline Gait, Cognitive and urinary scores: NR<br>Pre-op iNPH score (median): 5.5 (5-7)<br>**Index test and outcomes available in 38 patients**
9. **Gao 2025,**[9] China, Aug 2019-Nov 2023; Retrospective cohort study<br>Funding: Key R&D Program of Zhejiang Province, National Natural Science Foundation of China, A Project Supported by Scientific Research<br>Fund of Zhejiang University<br>Aim of the study: To study the predictive performance of preoperative imaging features combined with the tap test for the outcomes of ventriculoperitoneal (VP) shunt in idiopathic normal pressure hydrocephalus (iNPH).<br>Participants: Diagnostic criteria were based on the second edition of the Japanese iNPH Treatment Guidelines<br>Exclusions: All possible causes of secondary hydrocephalus; blurred magnetic resonance imaging (MRI) images<br>**CSF tap test was not used in the decision-making process for shunting**	211 patients; Mean age: 71(7); M/F: 110/56<br>Vascular disease: 44%; Cognitive impairment: 77%; Gait dysfunction: 97%; Urinary dysfunction: 79%<br>Duration of symptoms: 19(7.4 months)<br>Evans index: 0.35<br>Baseline scores: Not reported<br>CSF opening pressure: 12.9 cm in responders, 13.4 in non-responders<br>**Index test and outcomes available in 166 patients**
10. **Poudel 2026**;[10] India; 2019-2024; Ambispective cohort study<br>Funding: No funding received;<br>Aim of the study: To study if the diagnostic accuracy of the CSF tap test to predict outcomes of shunt surgery; to describe the short-term outcomes of patients who undergo shunt surgery and compare them with those who do not.<br>Participants: Patients who had probable iNPH according to the Japanese guidelines, with more than one of the specific triad of symptoms of NPH - gait disturbance, urinary disturbances, or cognitive impairment, were included. All patients had imaging evidence of hydrocephalus with an Evans ratio of >0.3 and imaging features of iNPH.<br>**CSF Tap test was not used in the decision-making process for shunting**	40 patients; Mean age: 67.2 ± 4.3 in responders; 58.9 ± 13.5 years in non responders; Vascular disease: NR<br>Gait dysfunction: 100%; cognitive impairment: 87-100%; Urinary dysfunction: 93-100%<br>Duration of symptoms: median 26 (IQR: 12–84) months in responders and 24 (IQR: 11–60) in non-responders<br>Pre-op iNPH score median 4(IQR 3-7); MoCA responders 20, non-responders 7. CSF opening pressure: NR<br>**Index test and outcomes available in 24 patients**

Supplementary Table 2: CSF Tap Test Diagnostic 2×2 Data for the included studies

Study	TP	FP	FN	TN	Total	CSF tap test positivity criteria (assessment timing)	Reference standard (shunt response) criteria
**Wikkelsö 1986**[1]	16	0	2	3	21	Walk test (steps to walk 18m); 5% improvement when assessed 2 h post-tap	Not reported
**Ishikawa 2012**[2]	74	16	6	4	100	≥1pt iNPHGS, OR >10% TUG, OR >3pt MMSE improvement; gait assessed 1–2d, cognition/urinary 1wk post-tap	≥1pt improvement on mRS; assessed at 12 months
**Wikkelsö 2013**[3]	49	7	45	10	111	≥5% mean improvement across motor/psychometric tests; assessed 3 h post-drainage (vs. baseline 24 h prior)	iNPH ≥5pt improvement OR mRS ↓1pt; assessed at 12 months postoperatively
**Yamada 2017**[4]	19	18	12	33	82	≥1pt improvement in total iNPHGS; gait assessed <24h, cognition <7d post-tap	Same outcome (total iNPHGS) improved; assessed at 12 months
**Liu 2021**[5]	9	4	7	3	23	Cumulative TUG: ≥10% reduction in time/steps at any of 8h/24h/72h post-LP	≥1pt increase in Japanese iNPHGS + patient/caregiver-reported improvement; assessed at 34±26 months post-surgery
**Rydja 2021**[6]	62	12	29	13	116	> 5 pt improvement in gait domain (Hellström iNPH scale); assessed ~3h post-LP	≥5pt improvement, total Hellström iNPH scale; assessed 3 months post-surgery
**Kameda 2022**[7]	6	4	20	10	40	TUG improvement ≥10%; assessed 1–3d post-tap (inpatient) or immediate–2wk (outpatient), best value used	Not specified (cutoff and timing not reported)
**vanBilsen 2022**[8]	20	9	6	3	38	No cutoffs reported; assessment timing not stated	≥1pt improvement in NPH score; median follow-up 6 months
**Gao 2025**[9]	61	28	35	42	166	≥20% improvement in 10m-walk test, and/or ≥10% improvement in MMSE, and/or ≥1pt improvement in urinary incontinence score; assessed 24–72h post-tap	Krauss index ≥0.5; assessed at 6–12 months follow-up
**Poudel 2026**[10]	8	3	7	6	24	Reduction of one or more points in mRS at 24 hours post CSF-TT	Reduction in one or more points in mRS at 24 weeks post shunt surgery
**Total**	324	101	169	127	721		

TP = CSF-TT positive, shunt responder; FP = CSF-TT positive, shunt non responder; FN = CSF-TT negative, Shunt responder; TN = CSF-TT negative, Shunt non-responder; MMSE: Mini mental state examination; mRS: modified Rankin scale; iNPHGS: iNPH gait scale

Supplementary Table 3: Demographics and Clinical Details of included studies reporting the diagnostic accuracy of the LIT

**Study ID; Country; Duration**<br>**Aims, Population description, Inclusion and exclusion criteria**	**Number of patients; Mean age; Clinical features, Evans index, baseline gait, urinary and cognitive scores**	**Plateau pressure, resistance to outflow, pulse pressure amplitude, baseline ICP**
**Boon 1997;** Netherlands;[11] September 1990-July 1995; Prospective cohort study<br>Funding: Doctor Ed. Hoelen Stichting and a foundation that wishes to remain anonymous<br>Aim of the study: To study if the measurement of resistance to outflow of cerebrospinal fluid (Rcsf) predicts outcome following surgery in iNPH<br>Participants: 1) a gradually developed gait disturbance of both legs, unexplained by other conditions, and a gait scale score of at least 12; 2) a mild-to-moderate cognitive deficit without aphasia, emerging with or after the gait disturbance, and a dementia scale score of at least 12; 3) a disability mRS grade of at least 2; and 4) a computerized tomography (CT) scan showing a communicating hydrocephalus with an Evans' index of 0.3 or greater and a ventricular index greater than 0.8,4 without clinically relevant parenchymal lesions, the sum of the four largest sulci at the convexity being less than 25 mm<br>Exclusions: Acute or subacute symptomatic NPH within 3 months of a causative incident, age of 85 years or more, severe comorbidity with restricted life expectancy, or contraindications for surgery	No of patients 101; Mean age: 74(6.3); Range M/F:60/41<br>Vascular disease; Cognitive impairment, Gait dysfunction; urinary dysfunction - NR<br>Duration of symptoms: 2.3±2.3 years Evans Index: 0.39±0.06<br>Baseline Gait score: 25.6±10.2<br>Baseline Cognitive score: 23.3±7.6; Baseline mRS: 3.7±1.3<br>Index test and outcomes available in 95 patients	Plateau pressure, Resistance to outflow, Pulse pressure amplitude, PA ICP ratio: NR<br>Baseline ICP 11.2±3.3
**Takeuchi 2000**; Japan;[12] July 95-Dec 97: Cohort - not clear if prospective or retrospective<br>Funding: Japanese Ministry of Health and Welfare grant-in-aid for scientific research into intractable hydrocephalus<br>Aim of the study: To study the effectiveness of shunt surgery in patients with iNPH with cerebral atrophy (atypical iNPH)<br>Participants: Idiopathic NPH presenting with Dementia and Brain atrophy; Inclusions: main complaint of dementia, sometimes with gait disturbance; intermediate or severe cerebral atrophy, bilateral symmetrical ventricular dilatation, and at least periventricular low density at the anterior horn on CT; and CSF pressure within the normal range with inflows of contrast medium into the cerebral ventricle or cortical surface 24 hours later on cisternography	No of patients: 25<br>Mean age: 60.4 (47-83); M/F: 15/10<br>Vascular disease: NR; Cognitive impairment, Gait dysfunction, urinary dysfunction: NR<br>Duration of symptoms: NR<br>Evans index and baseline scores: NR<br>Index test and reference standard outcomes available in 25	Plateau pressure: NR<br>Resistance to outflow: responder: 35.33 (11.16) non-responder: 9.12 (3.51)<br>Pulse pressure amplitude: NR; baseline ICP: NR; PA ICP ratio: NR; Pressure wave seen in 66.7% of shunt responders and 0% of shunt non-responders
**Sorteberg 2004**;[13] Norway; Prospective cohort study<br>Funding: Not reported<br>Aim of the study: To study the predictive value of the lumbar infusion test with computation of resistance to cerebrospinal fluid (CSF) outflow (Rout) and continuous ICP monitoring with computation of baseline or mean ICP and frequency of ICP elevations<br>Participants: Inclusions: Clinical signs of NPH, including gait disorder, urinary incontinence, and decreased memory; radiological findings of ventriculomegaly (Evans index >0.3)<br>Exclusions: Other diseases causing symptoms indicative of NPH; Non-idiopathic hydrocephalus; incomplete preoperative work-up.	No of patients 17; Mean age: 65; M/F: 7; Vascular disease: NR ; Cognitive impairment:88.2%<br>Gait dysfunction: 94%<br>Urinary dysfunction: 88.2%<br>Duration of symptoms: 1.5 years (6 months – 6 years; Evans index: >0.3 in all<br>Index test and reference standard outcomes available in 17	Plateau pressure: 37.14 +8.13 mmHg<br>Resistance to outflow: 16.55(4.05)(8.7-23.7) mmHg/ml/min<br>Baseline ICP: 8.75 ± 3.88 mmHg
**Eide 2005**;[14] Norway; April 2002-March 2003; Retrospective cohort<br>Funding: Not reported<br>Aim of the study: To analyze the preoperative spinal hydrodynamics during lumbar infusion tests of all iNPH cases treated with shunts and to evaluate to what extent these findings correlated to changes in the NPH score 1 year after shunt treatment.<br>Participants: Inclusions: Patients with the presence of minimum two deficits (gait disturbance, urinary incontinence, or dementia) and an Evans index > 0.3, treated with shunts	No of patients: 19. Mean age: 68 y (Range 47-81); M/F: 8/11. Vascular disease: 26%. Cognitive impairment, Gait dysfunction, Urinary dysfunction: NR. Duration of symptoms: 2.5 years (1-35). Evans Index: >0.3 in all; Preoperative iNPH 9 (5-14), postoperative iNPH 11 (4-15)<br>Index test and reference standard outcomes available in 19	Plateau pressure: NR. Resistance to outflow: median 16.3 mmHg/ml/min (ranges 4.6-21.0); Pulse pressure amplitude: baseline ICP: PA ICP ratio: NR. Mean CSFP wave amplitudes during LIT: significantly higher in shunt responders than in the non-responders.
**Hasselbalch 2023**;[15] Denmark; 2013-2020; Retrospective cohort<br>Funding: Danish Dementia Research Centre<br>Aim of the study: To investigate the predictive power of Rout, PA, and the PA/ICP ratio and to combine the best of the two with the iNPH Radscale in an attempt to increase the ability to predict short-term shunt response in patients with iNPH.<br>Participants: Patients with probable or possible NPH; Inclusions: (1) diagnosis of probable or possible iNPH according to international guideline criteria (2) lumbar infusion test as part of the diagnostic evaluation (3) an available brain CT or MRI scan, performed within 1 year prior to surgery (4) subsequent ventriculoperitoneal shunt (5) at least 2 months of clinical follow-up after operation	No of patients 127; Mean age: 74±6; Range 55-87 M/F: 46/81<br>Vascular disease: NR; Cognitive impairment, Gait dysfunction, urinary dysfunction: NR<br>Duration of symptoms: 3.7±3.1 years; Evans Index: Not reported<br>Baseline Gait score: 3.9±1.7(1-8)<br>Baseline Cognitive score: 25.2±4.1(11-30); Baseline urinary score: 2.9±1.2; (0-6)<br>Index test and reference standard outcomes available in 127	Plateau pressure: NR<br>Resistance to outflow: 15.9±6.5; 3.7-48.5; Pulse pressure amplitude: 3.8±1.9; Baseline ICP: 11.3±3.1; PA/ICP Ratio: 0.34 ± 0.17.
**Wikkelsö 2013**;[3] Details in Supplementary Table 1	Details in Supplementary Table 1<br>Index test and reference standard outcomes available in 110	Plateau pressure, Resistance to outflow, Pulse pressure amplitude, baseline ICP, PA ICP ratio NR

Supplementary Table 4: Lumbar Infusion Test (LIT) Diagnostic 2×2 Data of the included studies

Study	TP	FP	FN	TN	Total	Index test positivity criteria (assessment timing)	Reference standard (shunt response) criteria (assessment timing)
**Boon 1997[11]**	56	23	10	6	95	Rout ≥12 mmHg/mL/min*	≥mRS 1pt improvement (specific to this cutoff); assessed 1 month post-surgery
**Takeuchi 2000[12]**	11	1	1	12	25	Rout >20 mmHg/mL/min	iNPH grade improvement by ≥1; timing of surgery and outcome assessment not reported
**Sorteberg 2004[13]**	13	1	2	1	17	Rout >12 mmHg/mL/min	Not mentioned; outcomes assessed ~6 months postoperatively
**Eide 2005[14]**	7	6	1	5	19	Rout ≥12 mmHg/mL/min*	Bech iNPH score improvement ≥3; assessed 1 year post-shunt
**Wikkelsö 2013[3]**	65	11	28	6	110	Rout >12 mmHg/mL/min*	Hellström iNPH scale ≥5pt improvement OR mRS ≥1pt decrease; outcome data at 1 year
**Hasselbalch 2023[15]**	79	18	25	5	127	Rout ≥11 mmHg/mL/min* (25th percentile)	Summary shunt response score ≥1 (Hellström-based); assessed at mean of 7.4 months (range 2–20)
**Total**	231	60	67	35	393		

***For the pooled estimate calculation, this Rout threshold was selected from among the several thresholds tested in the study (see Table 2 in the manuscript for all cutoffs evaluated).**

TP = LIT positive, shunt responder; FP = LIT positive, shunt non responder; FN = LIT negative, Shunt responder; TN = LIT negative, Shunt non-responder

Rout: resistance to outflow; mRS: modified Rankin Scale

Supplementary Table 5: Leave-one-out sensitivity analysis for CSF-TT

Study left out	Sens (95% CI)	Spec (95% CI)	Tau² sens	I² sens (%)	Tau² spec	I² spec (%)	Gen Tau²	Gen I² (%)	Covariance	Correlation coefficient
**None (all studies included)**	66.23 (52.03–78.00)	54.49 (42.62–65.87)	0.76	79.96	0.37	47.26	0.00	0.04	-0.53	-1.00
**Wikkelsø 1986**[1]	64.05 (49.21–76.62)	54.80 (41.75–67.23)	0.73	81.28	0.44	59.74	0.00	0.02	-0.57	-1.00
**Ishikawa 2012**[2]	60.99 (49.47–71.41)	59.00 (50.49–67.00)	0.37	68.52	0.08	16.49	0.00	0.05	-0.17	-1.00
**Wikkelsø 2013**[3]	68.00 (52.63–80.25)	53.76 (40.76–66.26)	0.81	79.12	0.42	49.09	0.00	0.05	-0.58	-1.00
**Yamada 2017**[4]	66.94 (51.15–79.66)	52.40 (39.50–64.98)	0.86	81.13	0.39	46.53	0.00	0.11	-0.58	-1.00
**Liu 2021**[5]	66.94 (51.34–79.53)	54.64 (41.82–66.86)	0.85	82.53	0.41	51.42	0.00	0.05	-0.59	-1.00
**Rydja 2021**[6]	66.05 (49.91–79.16)	54.78 (41.72–67.21)	0.88	80.56	0.41	48.33	0.00	0.01	-0.60	-1.00
**Kameda 2022**[7]	70.10 (58.45–79.62)	51.62 (39.50–63.55)	0.46	71.69	0.33	44.38	0.00	0.00	-0.39	-1.00
**vanBilsen 2022**[8]	64.59 (48.96–77.62)	57.05 (45.45–67.93)	0.82	81.47	0.30	42.34	0.00	0.02	-0.50	-1.00
**Gao 2025**[9]	66.32 (50.22–79.36)	52.73 (39.25–65.82)	0.87	80.42	0.42	47.82	0.00	0.10	-0.60	-1.00
**Poudel 2026**[10]	67.52 (52.21–79.81)	53.34 (40.72–65.54)	0.82	82.27	0.39	49.37	0.00	0.01	-0.57	-1.00

Supplementary Table 6: Leave-one-out sensitivity analysis of LIT

Study left out	Sens (95% CI)	Spec (95% CI)	Tau² sens	I² sens (%)	Tau² spec	I² spec (%)	Gen Tau²	Gen I² (%)	Covariance	Correlation coefficient
**None (all studies included)**	77.47 (70.19–83.40)	28.27 (19.00–39.85)	0.05	14.69	0.03	4.57	0.00	0.00	-0.04	-1.00
**Boon 1997[11]**	74.55 (68.38–79.86)	32.08 (20.96–45.67)	0.00	0.00	0.00	0.00	0.00	0.00	-0.00	-1.00
**Sorteberg 2004[13]**	76.70 (69.33–82.74)	27.97 (18.43–40.03)	0.04	16.31	0.04	13.46	0.00	0.00	-0.04	-1.00
**Eide 2005[14]**	77.38 (69.87–83.46)	25.50 (16.16–37.80)	0.05	25.10	0.03	3.94	0.00	0.00	-0.04	-1.00
**Wikkelsø 2013[3]**	80.37 (71.97–86.72)	26.16 (16.91–38.14)	0.00	0.59	0.00	0.03	0.00	0.00	0.00	1.00
**Hasselbalch 2023[15]**	78.97 (68.17–86.81)	30.89 (19.32–45.47)	0.11	23.85	0.06	6.89	0.00	0.00	-0.08	-1.00

Supplementary Table 7: Risk of bias assessment QUADAS-2

Study ID	Domain 1: Patient selection: Risk of Bias<br>Applicability: Concern that the included patients do not match the review question	Domain 2: Index test - Risk of Bias<br>Applicability concerns: Is there a concern that the index test, its conduct, or its interpretation differs from the review question?	Domain 3: Reference standard - Risk of bias<br>Applicability concerns: Is there a concern that the target condition, as defined by the reference standard, does not match the review question?	Domain 4: Flow and timing - Risk of bias
**1. Wikkelsö 1986**[1]	Consecutive or random sample enrolled: Yes<br>Case-control avoided: Yes<br>Avoided inappropriate exclusions: No, only 21 patients underwent evaluation with a walk test, and only 24/27 had postoperative outcomes.<br>**Could the selection of patients have introduced bias? High**<br>**Applicability concerns: High** (post-SAH, head trauma included)	Index test interpreted without knowledge of reference standard: Yes<br>Threshold pre-specified: Yes<br>**Could the conduct or interpretation of the index test have introduced bias? Low**<br>**Applicability concerns: Low**	Reference standard likely to correctly classify the target condition: Unclear (no criteria)<br>Were the reference standard results interpreted without knowledge of the results of the index test? Unclear.<br>**Could the reference standard, its conduct, or its interpretation have introduced bias? Unclear**<br>**Applicability concerns: No**	Appropriate interval between index test and reference standard: Unclear<br>Did all patients receive a reference standard? No<br>Did patients receive the same reference standard? Yes<br>Were all patients included in the analysis? No<br>**Could the patient flow have introduced bias? High**
**2. Ishikawa 2012**[2]	Consecutive or random sample enrolled: Yes<br>Case-control avoided: Yes<br>Avoided inappropriate exclusions: Yes<br>**Could the selection of patients have introduced bias? Low**<br>**Applicability Concerns: Low**	Index test interpreted without knowledge of reference standard: Yes<br>Threshold pre-specified: Yes.<br>**Could the conduct or interpretation of the index test have introduced bias? Low**<br>**Applicability concerns: Low**	Reference standard likely to correctly classify the target condition: Yes<br>Were the reference standard results interpreted without knowledge of the results of the index test? Unclear<br>**Could the reference standard, its conduct, or its interpretation have introduced bias? Unclear**<br>**Applicability concerns: Low**	Appropriate interval between index test and reference standard: Yes (done within 2 months)<br>Did all patients receive a reference standard? Yes<br>Did patients receive the same reference standard? Yes<br>Were all patients included in the analysis? Yes<br>**Could the patient flow have introduced bias? Low**
**3. Wikkelsö 2013**[3]	Consecutive or random sample enrolled: Yes<br>Case-control avoided: Yes<br>Avoided inappropriate exclusions: No (115/142 only had outcomes at 1 y)<br>**Could the selection of patients have introduced bias? High**<br>**Applicability Concerns: Low**	Index test interpreted without knowledge of reference standard: Yes<br>Threshold pre-specified: Yes<br>**Could the conduct or interpretation of the index test have introduced bias? Low**<br>**Applicability concerns: Low**	Reference standard likely to correctly classify the target condition: Yes<br>Were the reference standard results interpreted without knowledge of the results of the index test? Yes<br>**Could the reference standard, its conduct, or its interpretation have introduced bias? Low**<br>**Applicability concerns: Low**	Appropriate interval between index test and reference standard: Unclear<br>Did all patients receive a reference standard? Yes<br>Did patients receive the same reference standard? Yes<br>Were all patients included in the analysis?No<br>**Could the patient flow have introduced bias? High**
**4. Yamada 2017**[4]	Consecutive or random sample enrolled: Yes<br>Case-control avoided: Yes<br>Avoided inappropriate exclusions: No (82/93 had final data available)<br>**Could the selection of patients have introduced bias? High**<br>**Applicability Concerns: Low**	Index test interpreted without knowledge of reference standard: Yes<br>Threshold pre-specified: Yes<br>**Could the conduct or interpretation of the index test have introduced bias? Low**<br>**Applicability concerns: Low**	Reference standard likely to correctly classify the target condition: Yes<br>Were the reference standard results interpreted without knowledge of the results of the index test? Unclear<br>**Could the reference standard, its conduct, or its interpretation have introduced bias? Unclear**<br>**Applicability concerns: Low**	Appropriate interval between index test and reference standard: Unclear<br>Did all patients receive a reference standard? Yes<br>Did patients receive the same reference standard? Yes<br>Were all patients included in the analysis?No<br>**Could the patient flow have introduced bias? High**

**5.**	**Liu 2021[5]**	Consecutive or random sample of patients enrolled: Yes<br>Case-control avoided: Yes<br>Study avoided inappropriate exclusions: No (only 88 patients underwent the index test, and only 23 of 115 patients admitted for evaluation had outcome data available).<br>**Could the selection of patients have introduced bias? High**<br>**Applicability concerns: Low**	Index test interpreted without knowledge of reference standard: Yes<br>Threshold pre-specified: Yes<br>**Could the conduct or interpretation of the index test have introduced bias? Low**<br>**Applicability concerns: Low**	Reference standard likely to correctly classify the target condition: Yes<br>Were the reference standard results interpreted without knowledge of the results of the index test? Unclear<br>**Could the reference standard, its conduct, or its interpretation have introduced bias? Unclear**<br>**Applicability concerns: Low**	Appropriate interval between index test and reference standard: Yes (Done within one month)<br>Did all patients receive a reference standard?No<br>Did patients receive the same reference standard? Yes<br>Were all patients included in the analysis?No<br>**Could the patient flow have introduced bias? High**
**6.**	**Rydja 2021[6]**	Consecutive or random sample of patients enrolled: No; Out of 159 iNPH patients, 116 patients (95 patients with probable iNPH and 21 with possible iNPH) were included in the statistical analysis. 43 were excluded: 25 did not undergo the CSF TT, five had been investigated with external lumbar drainage (ELD), 13 patients had no result from the follow-up postoperative assessment (six had shunt complications, five had missing data due to unwillingness to participate, and two died). One of the deaths was caused by an acute subdural hematoma 2 months after shunt surgery, and the other by an intracerebral hematoma 5 days after surgery.<br>Case-control avoided: Yes.<br>Avoided inappropriate exclusions: No.<br>**Could the selection of patients have introduced bias? High**<br>**Applicability concerns: Low**	Index test interpreted without knowledge of reference standard: Yes<br>Threshold pre-specified: Yes<br>**Could the conduct or interpretation of the index test have introduced bias? Low**<br>**Applicability concerns: Low**	Reference standard likely to correctly classify the target condition: Yes<br>Were the reference standard results interpreted without knowledge of the results of the index test? Unclear<br>**Could the reference standard, its conduct, or its interpretation have introduced bias? Unclear**<br>**Applicability: Low**	Appropriate interval between index test and reference standard: Unclear (Surgery was done 126 (102-159) days after baseline assessment)<br>Did all patients receive a reference standard?No<br>Did patients receive the same reference standard? Yes<br>Were all patients included in the analysis?No<br>**Could the patient flow have introduced bias? High**
**7.**	**Kameda 2022[7]**	Consecutive or random sample enrolled: Yes<br>Case-control avoided: Yes<br>Avoided inappropriate exclusions: Yes<br>**Could the selection of patients have introduced bias? Low**<br>**Applicability concerns: Low**	Index test interpreted without knowledge of reference standard: Yes<br>Threshold pre-specified: Yes<br>**Could the conduct or interpretation of the index test have introduced bias? Low**<br>**Applicability concerns: Low**	Reference standard likely to correctly classify the target condition: Yes<br>Were the reference standard results interpreted without knowledge of the results of the index test? Unclear<br>**Could the reference standard, its conduct, or its interpretation have introduced bias? Unclear**<br>**Applicability concerns: Low**	Appropriate interval between index test and reference standard: Unclear<br>Did all patients receive a reference standard? Yes<br>Did patients receive the same reference standard? Yes<br>Were all patients included in the analysis? Yes<br>**Could the patient flow have introduced bias? Unclear**
**8.**	**vanBilsen 2022[8]**	Consecutive or random sample enrolled: Yes<br>Case-control avoided: Yes<br>Avoided inappropriate exclusions: Yes<br>**Could the selection of patients have introduced bias? Low**<br>**Applicability concerns: Low**	Index test interpreted without knowledge of reference standard: Yes<br>Threshold pre-specified: No<br>**Could the conduct or interpretation of the index test have introduced bias? High**<br>**Applicability concerns: Unclear**<br>(CSF tap test cutoffs not mentioned for improvement)	Reference standard likely to correctly classify the target condition: Yes<br>Were the reference standard results interpreted without knowledge of the results of the index test? Yes<br>**Could the reference standard, its conduct, or its interpretation have introduced bias? Low**<br>**Applicability: Low**	Appropriate interval between index test and reference standard: Yes (1-3.5 months)<br>Did all patients receive a reference standard? Yes<br>Did patients receive the same reference standard? Yes<br>Were all patients included in the analysis? Yes

				**Could the patient flow have introduced bias? Low**
**9. Gao 2025[9]**	Consecutive or random sample enrolled: Yes<br>Case-control avoided: Yes<br>Avoided inappropriate exclusions: No (33 patients with incomplete data and MRI excluded; 12 patients were lost to follow-up)<br>**Could the selection of patients have introduced bias? Yes**<br>**Applicability concerns: Low**	Index test interpreted without knowledge of reference standard: Yes<br>Threshold pre-specified: Yes<br>**Could the conduct or interpretation of the index test have introduced bias? No**<br>**Applicability concerns: Low**	Reference standard likely to correctly classify the target condition: Yes<br>Were the reference standard results interpreted without knowledge of the results of the index test? Unclear<br>**Could the reference standard, its conduct, or its interpretation have introduced bias? Unclear**<br>**Applicability Concerns: Low**	Appropriate interval between index test and reference standard: Unclear<br>Did all patients receive a reference standard?No<br>Did patients receive the same reference standard? Yes<br>Were all patients included in the analysis? No<br>**Could the patient flow have introduced bias? High**
**10. Poudel 2026[10]**	Consecutive or random sample enrolled: Yes<br>Case-control avoided: Yes<br>Avoided inappropriate exclusions: Yes<br>**Could the selection of patients have introduced bias? No**<br>**Applicability concerns: Low**	Index test interpreted without knowledge of reference standard: Yes<br>Threshold pre-specified: Yes<br>**Could the conduct or interpretation of the index test have introduced bias? No**<br>**Applicability concerns: Low**	Reference standard likely to correctly classify the target condition: Yes<br>Were the reference standard results interpreted without knowledge of the results of the index test? No<br>**Could the reference standard, its conduct, or its interpretation have introduced bias? Yes**<br>**Applicability Concerns: Low**	Appropriate interval between index test and reference standard: Yes<br>Did all patients receive a reference standard? Yes<br>Did patients receive the same reference standard? Yes<br>Were all patients included in the analysis? Yes<br>**Could the patient flow have introduced bias? No**
**11. Boon 1997[11]**	Consecutive or random sample enrolled: Yes<br>Case-control avoided: Yes<br>Avoided inappropriate exclusions: No (6 patients who died before the first follow-up were excluded)<br>**Could the selection of patients have introduced bias? Yes**<br>**Applicability concerns: Low**	Index test interpreted without knowledge of reference standard: Yes<br>Threshold pre-specified: Yes<br>**Could the conduct or interpretation of the index test have introduced bias? No**<br>**Applicability concerns: Low**	Reference standard likely to correctly classify the target condition: Yes<br>Were the reference standard results interpreted without knowledge of the results of the index test? Unclear<br>**Could the reference standard, its conduct, or its interpretation have introduced bias? Unclear**<br>**Applicability Concerns: Low**	Appropriate interval between index test and reference standard: Unclear<br>Did all patients receive a reference standard? Yes<br>Did patients receive the same reference standard? Yes<br>Were all patients included in the analysis? No<br>**Could the patient flow have introduced bias? High**
**12. Takeuchi 2000[12]**	Consecutive or random sample enrolled: Yes<br>Case-control avoided: Yes<br>Avoided inappropriate exclusions: Yes<br>**Could the selection of patients have introduced bias? Low**<br>**Applicability Concerns: Unclear** (Included only patients with atypical iNPH)	Index test interpreted without knowledge of reference standard: Yes<br>Threshold pre-specified: Unclear (Ro cutoff of 20 was taken; it is not clear if it was decided a priori);<br>**Could the conduct or interpretation of the index test have introduced bias? Unclear**<br>**Applicability concerns: High** (Ro of >=20 is not standard as per literature)	Reference standard likely to correctly classify the target condition: Yes<br>Were the reference standard results interpreted without knowledge of the results of the index test? Unclear<br>**Could the reference standard, its conduct, or its interpretation have introduced bias? Unclear**<br>**Applicability Concerns: Low concerns**	Appropriate interval between index test and reference standard: Unclear<br>Did all patients receive a reference standard? Yes<br>Did patients receive the same reference standard? Yes<br>Were all patients included in the analysis? Yes<br>**Could the patient flow have introduced bias? Unclear**
**13. Sorteberg 2004[13]**	Consecutive or random sample enrolled: Yes<br>Case-control avoided: Yes<br>Avoided inappropriate exclusions: Yes<br>**Could the selection of patients have introduced bias? Low**<br>**Applicability Concerns: Low**	Index test interpreted without knowledge of reference standard: Yes<br>Threshold pre-specified: Yes.<br>**Could the conduct or interpretation of the index test have introduced bias? Low**<br>**Applicability concerns: Low**	Reference standard likely to correctly classify the target condition: Unclear<br>Were the reference standard results interpreted without knowledge of the results of the index test? Unclear.	Appropriate interval between index test and reference standard: Unclear<br>Did all patients receive a reference standard? Yes<br>Did patients receive the same reference standard? Yes<br>Were all patients included in the analysis? Yes

			**Could the reference standard, its conduct, or its interpretation have introduced bias? Unclear**<br><br>**Applicability concerns: Low**	**Could the patient flow have introduced bias? Unclear**
**14. Eide 2005**[14]	Consecutive or random sample enrolled: Yes<br><br>Case-control avoided: Yes<br><br>Avoided inappropriate exclusions: No (Even though clinical and radiologic findings were used to select patients for shunt surgery, Rout values in 10 other subjects evaluated for possible NPH and not receiving a shunt during the same study period were lower (median value 10.5 mmHg/ml/min, ranges 6.9 – 14.5 mmHg/ml/min). There was, hence, a certain referral bias regarding preoperative Rout values and selection for shunt surgery.)<br><br>**Could the selection of patients have introduced bias? High**<br><br>**Applicability Concerns: Unclear**	Index test interpreted without knowledge of reference standard: Yes<br><br>Threshold pre-specified: Unclear (No specific mention made, but Rout 12 is the standard literature-suggested threshold)<br><br>**Could the conduct or interpretation of the index test have introduced bias? Unclear**<br><br>**Applicability concerns: Low**	Reference standard likely to correctly classify the target condition: Yes<br><br>Were the reference standard results interpreted without knowledge of the results of the index test? Unclear<br><br>**Could the reference standard, its conduct, or its interpretation have introduced bias? Unclear**<br><br>**Applicability concerns: Low**	Appropriate interval between index test and reference standard: Yes (A ventriculo-peritoneal (VP) shunt was implanted 2 - 3 weeks)<br><br>Did all patients receive a reference standard? Yes<br><br>Did patients receive the same reference standard? Yes<br><br>Were all patients included in the analysis? Yes<br><br>**Could the patient flow have introduced bias? Low**
**15. Hasselbalch 2023**[15]	Consecutive or random sample enrolled: Yes<br><br>Case-control avoided: Yes<br><br>Avoided inappropriate exclusions: Unclear, "361 consecutive patients diagnosed with possible or probable iNPH. 127 patients met the inclusion criteria. Only patients who had outcomes of at least 2 months were included. Out of 361, only 127 patients were included in the analysis) (Unclear if all clinico-radiologically diagnosed patients were selected to be enrolled in the study)<br><br>**Could the selection of patients have introduced bias? Unclear**<br><br>**Applicability concerns: Unclear**	Index test interpreted without knowledge of reference standard: Yes<br><br>Threshold pre-specified: No<br><br>**Could the conduct or interpretation of the index test have introduced bias? High**<br><br>**Applicability concerns: Low**	Reference standard likely to correctly classify the target condition: No (Due to the retrospective design of the study, postoperative objective measures for gait and cognition (10-m gait test and MMSE) were only available in 70% and 80% of the patients, respectively.<br><br>Were the reference standard results interpreted without knowledge of the results of the index test? Unclear.<br><br>**Could the reference standard, its conduct, or its interpretation have introduced bias? High**<br><br>**Applicability Concerns: High**	Appropriate interval between index test and reference standard: Unclear<br><br>Did all patients receive a reference standard?No<br><br>Did patients receive the same reference standard? Yes<br><br>Were all patients included in the analysis?No<br><br>**Could the patient flow have introduced bias? High**

Supplementary Table 8: GRADE summary of findings table for the CSF-TT

Should the CSF tap test be used to screen for shunt responsiveness in idiopathic normal pressure hydrocephalus?

Sensitivity	0.64 (95% CI: 0.49 to 0.77)
Specificity	0.55 (95% CI: 0.42 to 0.67)

Prevalences	50%	60%	80%

Outcome	№ of studies (№ of patients)	Study design	Factors that may decrease the certainty of evidence					Effect per 1,000 patients tested			Test accuracy CoE
			Risk of bias	Indirectness	Inconsistency	Imprecision	Publication bias	Pre-test probability of 50%	Pre-test probability of 60%	Pre-test probability of 80%	
**True positives** (patients with shunt responsiveness)	9 studies 700 patients	cross-sectional (cohort type accuracy study)	serious[a]	not serious	very serious[b]	serious[c]	none	320 (245 to 385)	384 (294 to 462)	512 (392 to 616)	⊕◯◯◯ Very low[a,b,c]
**False negatives** (patients incorrectly classified as not having shunt responsiveness)								180 (115 to 255)	216 (138 to 306)	288 (184 to 408)	
**True negatives** (patients without shunt responsiveness)	9 studies 700 patients	cross-sectional (cohort type accuracy study)	serious[a]	not serious	serious[d]	not serious[e]	none	275 (210 to 335)	220 (168 to 268)	110 (84 to 134)	⊕⊕◯◯ Low[a,d,e]
**False positives** (patients incorrectly classified as having shunt responsiveness)								225 (165 to 290)	180 (132 to 232)	90 (66 to 116)	

Explanations

a. High risk of bias for patient selection, flow, and timing domains

b. Point estimates are all over the place, with high statistical heterogeneity

c. The confidence intervals are crossing a clinically meaningful threshold of 70% sensitivity, but the upper limit of the confidence interval is 77%, showing possible usefulness

d. Considering a clinically meaningful specificity threshold of 70%, most studies fall below this threshold with substantial statistical heterogeneity

e. Considering a specificity of 70% to be clinically meaningful, both sides of the confidence interval are below this threshold, thus providing a precise estimate that the test is poorly specific

Supplementary Table 9: GRADE summary of findings table for the LIT

Should the lumbar infusion test be used to screen for shunt responsiveness in idiopathic normal pressure hydrocephalus?

Sensitivity	0.77 (95% CI: 0.70 to 0.83)
Specificity	0.28 (95% CI: 0.19 to 0.40)

Prevalences	50%	60%	80%

Outcome	№ of studies (№ of patients)	Study design	Factors that may decrease the certainty of evidence					Effect per 1,000 patients tested			Test accuracy CoE
			Risk of bias	Indirectness	Inconsistency	Imprecision	Publication bias	Pre-test probability of 50%	Pre-test probability of 60%	Pre-test probability of 80%	
**True positives** (patients with shunt responsiveness)	5 studies 368 patients	cross-sectional (cohort type accuracy study)	serious[a]	not serious	not serious	not serious	none	385 (350 to 415)	462 (420 to 498)	616 (560 to 664)	⨁⨁⨁◯ Moderate[a]
**False negatives** (patients incorrectly classified as not having shunt responsiveness)								115 (85 to 150)	138 (102 to 180)	184 (136 to 240)	
**True negatives** (patients without shunt responsiveness)	5 studies 368 patients	cross-sectional (cohort type accuracy study)	serious[a]	not serious	not serious	not serious[b]	none	140 (95 to 200)	112 (76 to 160)	56 (38 to 80)	⨁⨁⨁◯ Moderate[a,b]
**False positives** (patients incorrectly classified as having shunt responsiveness)								360 (300 to 405)	288 (240 to 324)	144 (120 to 162)	

Explanations

a. High risk of bias for patient selection and flow and timing domains

b. Considering a specificity of 70% to be clinically meaningful, both sides of the confidence interval are below this threshold, thus providing a precise result that the test is not specific

References

1. Wikkelsö C, Andersson H, Blomstrand C. Normal pressure hydrocephalus. Predictive value of the cerebrospinal fluid tap-test. *Acta Neurol Scand*. 1986;73(6):566-573.

2. Ishikawa M, Hashimoto M, Mori E, Kuwana N, Kazui H. The value of the cerebrospinal fluid tap test for predicting shunt effectiveness in idiopathic normal pressure hydrocephalus. *Fluids Barriers CNS*. 2012;9(1). doi:10.1186/2045-8118-9-1

3. Wikkelsö C, Hellström P, Klinge PM, Tans JTJ. The European iNPH Multicentre Study on the predictive values of resistance to CSF outflow and the CSF Tap Test in patients with idiopathic normal pressure hydrocephalus. *J Neurol Neurosurg Psychiatry*. 2013;84(5):562-568. doi:10.1136/jnnp-2012-303314

4. Yamada S, Ishikawa M, Miyajima M, et al. Disease duration: the key to accurate CSF tap test in iNPH. *Acta Neurol Scand*. 2017;135(2):189-196. doi:10.1111/ane.12580

5. Liu C, Dong L, Li J, et al. A pilot study of multiple time points and multidomain assessment in cerebrospinal fluid tap test for patients with idiopathic normal pressure hydrocephalus. *Clin Neurol Neurosurg*. 2021;210((Liu C.; Dong L.; Li J.; Huang X.; Wang J.; Lei D.; Mao C.; Cui L.; Gao J., gj107@163.com) Department of Neurology, State Key Laboratory of Complex Severe and Rare Disease, Peking Union Medical College Hospital, Chinese Academy of Medical Sciences, Beijin). doi:10.1016/j.clineuro.2021.107012

6. Rydja J, Eleftheriou A, Lundin F. Evaluating the cerebrospinal fluid tap test with the Hellström iNPH scale for patients with idiopathic normal pressure hydrocephalus. *Fluids Barriers CNS*. 2021;18(1). doi:10.1186/s12987-021-00252-5

7. Kameda M, Kajimoto Y, Kambara A, et al. Evaluation of the Effectiveness of the Tap Test by Combining the Use of Functional Gait Assessment and Global Rating of Change. *Front Neurol*. 2022;13((Kameda M., mrkameda@gmail.com; Kajimoto Y., yoshinaga.kajimoto@ompu.ac.jp; Kambara A.; Tsujino K.; Yamada H.; Takagi F.; Fukuo Y.; Kosaka T.; Kanemitsu T.; Katayama Y.; Tsuji Y.; Yagi R.; Hiramatsu R.; Ikeda N.; Nonoguchi N.; Furuse M.; Kawabata S.; Taka). doi:10.3389/fneur.2022.846429

8. van Bilsen MWT, van den Abbeele L, Volovici V, Boogaarts HD, Bartels RHMA, van Lindert EJ. The diagnostic value of the pulsatility curve to predict shunt responsiveness in patients with idiopathic normal pressure hydrocephalus. *Acta Neurochir (Wien)*. 2022;164(7):1747-1754. doi:10.1007/s00701-022-05233-7

9. Gao W, Liu W, Ying Y, et al. Preoperative imaging biomarkers combined with tap test for predicting shunt surgery outcome in idiopathic normal pressure hydrocephalus: a multicenter retrospective study. *Front Aging Neurosci*. 2025;17. doi:10.3389/fnagi.2025.1509493

10. Poudel S, Dash D, Fasano A, et al. Diagnostic accuracy of CSF tap-test parameters in predicting shunt responsiveness in normal pressure hydrocephalus: a cohort study. *BMC Neurol*. Published online July 28, 2026. doi:10.1186/s12883-026-05151-6

11. Boon AJW, Tans JTJ, Delwel EJ, et al. Dutch Normal-Pressure Hydrocephalus Study: prediction of outcome after shunting by resistance to outflow of cerebrospinal fluid. *J Neurosurg*. 1997;87(5):687-693. doi:10.3171/jns.1997.87.5.0687

12. Takeuchi T, Kasahara E, Iwasaki M, Mima T, Mori K. Indications for Shunting in Patients with Idiopathic Normal Pressure Hydrocephalus Presenting with Dementia and Brain Atrophy (Atypical Idiopathic Normal Pressure Hydrocephalus). *Neurol Med Chir (Tokyo)*. 2000;40(1):38-47. doi:10.2176/nmc.40.38

13. Sorteberg A, Eide PK, Fremming AD. A prospective study on the clinical effect of surgical treatment of normal pressure hydrocephalus: The value of hydrodynamic evaluation. *Br J Neurosurg*. 2004;18(2):149-157. doi:10.1080/02688690410001681000

14. Eide PK, Sorteberg W. Preoperative spinal hydrodynamics versus clinical change 1 year after shunt treatment in idiopathic normal pressure hydrocephalus patients. *Br J Neurosurg*. 2005;19(6):475-483. doi:10.1080/02688690500495125

15. Hasselbalch SG, Carlsen JF, Alaouie MM, et al. Prediction of shunt response in idiopathic normal pressure hydrocephalus by combined lumbar infusion test and preoperative imaging scoring. *Eur J Neurol*. Published online 2023. doi:10.1111/ene.15981
