# Supplementary material for: Accuracy of CSF tap test and lumbar infusion test in predicting shunt response in idiopathic normal pressure hydrocephalus: a systematic review and meta-analysis
Source: Front Neurol. 2026 Oct 12;17:1908328. doi: 10.3389/fneur.2026.1908328 (PMC13710815; doi:10.3389/fneur.2026.1908328)
Supplement: Supplementary file 3 [file Supplementary_file_2.pdf]

Search strategy

Pubmed/Web of Science/Scopus

#1 (((normal pressure hydrocephalus) OR (NPH)) OR (iNPH)) OR (idiopathic normal pressure hydrocephalus)
#2 (((CSF tap test) OR (large volume CSF tap) OR (magnetic resonance imaging)) OR (MRI)) OR (lumbar infusion test)
#3 Ventriculoperitoneal shunt OR ventriculo peritoneal shunt OR shunt surgery OR CSF diversion procedure OR lumbar drain OR lumboperitoneal shunt OR thecoperitoneal shunt OR theco peritoneal shunt
((#1) AND (#2)) AND (#3)

Cochrane central

#1 Normal Pressure Hydrocephalus
#2 Idiopathic Normal Pressure Hydrocephalus
#3 NPH
#4 iNPH
#5 #1 OR #2 OR #3 OR #4
#6 CSF tap test
#7 Lumbar infusion test
#8 large volume CSF tap
#9 MRI
#10 Magnetic Resonance Imaging
#11 #6 OR #7 OR #8 OR #9 OR #10
#12 Ventriculoperitoneal shunt
#13 Ventriculo peritoneal shunt
#14 Shunt surgery
#15 CSF diversion procedure
#16 lumbar drain
#17 lumboperitoneal shunt
#18 thecoperitoneal shunt
#19 theco peritoneal shunt
#20 #12 OR #13 OR #14 OR #15 OR #16 OR #17 OR #18 OR #19
#21 #5 AND #11 AND #20

EMBASE

#1 Normal Pressure Hydrocephalus
#2 Idiopathic Normal Pressure Hydrocephalus
#3 NPH
#4 iNPH
#5 #1 OR #2 OR #3 OR #4
#6 CSF tap test
#7 Lumbar infusion test
#8 large volume CSF tap
#9 MRI
#10 Magnetic Resonance Imaging
#11 #6 OR #7 OR #8 OR #9 OR #10
#12 Ventriculoperitoneal shunt
#13 Ventriculo peritoneal shunt
#14 Shunt surgery

#15 CSF diversion procedure
#16 lumbar drain
#17 lumboperitoneal shunt
#18 thecoperitoneal shunt
#19 theco peritoneal shunt
#20 #12 OR #13 OR #14 OR #15 OR #16 OR #17 OR #18 OR #19
#21 #5 AND #11 AND #20
